# Knowledge about, attitude toward, and practice of complementary and alternative medicine among nursing students: A systematic review of cross-sectional studies

**DOI:** 10.3389/fpubh.2022.946874

**Published:** 2022-08-04

**Authors:** Fei-Yi Zhao, Gerard A. Kennedy, Sonja Cleary, Russell Conduit, Wen-Jing Zhang, Qiang-Qiang Fu, Zhen Zheng

**Affiliations:** ^1^Department of Nursing, School of International Medical Technology, Shanghai Sanda University, Shanghai, China; ^2^School of Health and Biomedical Sciences, RMIT University, Bundoora, VIC, Australia; ^3^Shanghai Municipal Hospital of Traditional Chinese Medicine, Shanghai University of Traditional Chinese Medicine, Shanghai, China; ^4^Institute of Health and Wellbeing, Federation University, Mount Helen, VIC, Australia; ^5^Institute for Breathing and Sleep, Austin Health, Heidelberg, VIC, Australia; ^6^Yangpu Hospital, School of Medicine, Tongji University, Shanghai, China

**Keywords:** complementary and alternative medicine, nursing students, nursing education, knowledge, attitude, practice, KAP, systematic review

## Abstract

**Background:**

The globally growing demand for complementary and alternative medicine (CAM) has attracted educators' attention to integrate CAM into conventional nursing programs. This systematic review aimed to understand the status quo of nursing students (NSs)' overall rated knowledge of, attitude/belief toward, and practice/previous use or experience (KAP) of CAM in surveys, given these factors may influence NSs' receptivity to CAM curricula, and may be of value in guiding the development of effective teaching strategies.

**Methods:**

Formally published cross-sectional quantitative studies investigating the primary outcome of KAP toward CAM by NSs were searched for from eight databases from their inception through to 28 April 2022. *PRISMA 2020* guidelines were followed.

**Results:**

Twenty-six studies were included for analysis, 25 of which were judged to be of moderate to high quality. Despite limited and poorly informed knowledge of CAM therapies, the majority of NSs generally viewed them in a positive light. Furthermore, NSs usually reported an interest in further learning, and supported and welcomed the integration of CAM curricula, at least as elective modules, into existing nursing programs. Lack of evidence was perceived as a major barrier to the use or integration of CAM. Mass media and the internet were the main sources *via* which NSs access CAM information. Measurement of KAP in all included studies was *via* self-designed questionnaires/scales or adapted from previously developed questionnaires/scales.

**Conclusions:**

The need for integrating and strengthening CAM curricula into current nursing education is identified. Besides theoretical knowledge and matched clinical placement, skills training in literature searching and evidence-based practice are advised to be included in the curricula design. The experiential learning mode is strongly recommended for delivering specific CAM modalities. In addition, a standard instrumentation for determining NSs' KAP toward CAM should be designed and examined for use in different cultural settings.

**Systematic Review Registration:**

https://www.crd.york.ac.uk/PROSPERO/display_record.php?RecordID=300602, identifier: PROSPERO CRD42022300602.

## Introduction

Complementary and alternative medicine (CAM) refers to an array of medical- and health- related systems, practices, and products that are not generally considered part of biomedical focused mainstream or conventional healthcare (1, 2). In accordance with the National Center for Complementary and Integrative Health (updated on April 2021), CAM interventions can be classified into five categories depending on their primary therapeutic input: nutritional (e.g., dietary supplements, special diets), psychological (e.g., hypnosis, mindfulness), physical (e.g., massage, spinal manipulation), combinations such as psychological and physical (e.g., dance, yoga) or psychological and nutritional (e.g., mindful eating) interventions, and other complementary health approaches (Appendix 1) (3). Despite the availability and benefits of modern biomedical models of healthcare, the demand for CAM therapies remains widespread and has been grown over the past decades (2, 4). The evidence base for use of certain CAM therapies is also expanding (5). A national population-based survey in 2007 revealed that 68.9% Australians had utilized at least one CAM therapy in the past 12 months; and the annual “out of pocket” expenditure on CAM was estimated over 4 billion Australian dollars (6). A similar survey in the same year in USA demonstrated that approximately four out of 10 adults and one out of nine children used CAM therapies in the previous 12 months (7). In European countries, it is estimated that 20–50% of the population utilized CAM therapies (8), and this percentage is even higher in Africa and Asia, where it is approximately 80% (9).

The evidence associated with the efficacy and safety of CAM is mixed, with some modalities widely accepted by mainstream healthcare whereas others remain controversial (10). For instance, diet and exercise therapies are considered to be a part of mainstream medical and healthcare practice (11); while, the usage of herbal products in combination with conventional medicine is poorly understood and considered to be risky (10). Worryingly, for a diversity of reasons (e.g., their perceptions that the medical physicians lack relevant knowledge, fear of being admonished or evoking negative responses, etc.), more than half of patients did not discuss or inform their physicians about experiences with CAM utilization, which increases lots of associated risks (10). Patient-provider communication about CAM approaches can support patient self-management by minimizing risk and optimizing care (12). In comparison with medical practitioners or other health professionals, nurses may play a more crucial part in communicating with patients about CAM utilization (13) and may be in a unique position to bridge the gap in communication between patients and their physicians, and between the clinical use of CAM and conventional medicine (14, 15). The rationale and justification for this supposition is as follows: Firstly, the basic tenets of most CAM approaches are individualization, and holistic in nature, that is to see humans as the totality of the biological, psychological and spiritual dimensions (8, 15–17). Those approaches are also the basis of many nursing practices (8, 15, 16). Secondly, nurses are the main providers of hands-on healthcare and the largest health professional group (17). Nurses typically spend more time building trust with patients and therefore patients usually feel more comfortable in disclosing details of CAM use to them (10). Thirdly, due to their predominant position in patient care, assessment, education, advocacy, and decision-making, nurses' knowledge of and attitudes about CAM therapies are also likely to affect patients' health beliefs, behaviors and decisions concerning these therapies (2, 17). It is particularly critical for nurses to provide or direct patients toward more evidence-based CAM information for those who rely on advertising or guidance from family and friends (10).

Accumulated evidence indicates that nurses with basic CAM knowledge have a higher likelihood of engaging patients in conversations about their use of CAM products and practice (10, 18–20). Conversely, lack of considerable knowledge is perceived by many nurses as a primary barrier to initiating CAM dialogue with patients (2, 10). More importantly, incorporating CAM into medical training has been found to significantly increase the safe use of integrative approaches in conventional healthcare (21). Nursing students (NSs), as future healthcare professionals, must be prepared to advise and guide patients to credible resources among a vast array of remedy options available and widely utilized (16), which is an embodiment of the patient-centered care mode. Furthermore, NSs' knowledge and perception of CAM are also key determinants to safe practice (9). This is because NSs' receptiveness to CAM can play a role in how CAM can be safely practiced alongside conventional medicine and if patients' need for CAM is considered in future medicine (22). Given the growing and continuing consumer demand for CAM worldwide (8, 16, 18), NSs thus should also be well-informed about some most commonly-used CAM therapies to the point where they can competently provide patients with holistic care, instruct patients to use some CAM therapies accurately and safely (4, 8), assess patients' usage of these therapies (16), and/or provide targeted referral services (18). After all, in addition to improving safety, equipping nurses with greater CAM knowledge would result in better and more evidence-based referrals to CAM services and thereupon increase the likelihood of patients receiving therapeutic interventions to ameliorate pathological conditions and improve quality of life (18). Unfortunately, whilst the nursing profession is rooted in an integrated practice that encompasses holism, caring and healing, existing nursing education programs do not always emphasize these approaches, particularly pathways for referral (16). Countries such as Australia (23), Turkey (8), and South Africa (13) rarely integrate CAM instruction/knowledge into their current nursing curricula/programs. In addition, CAM appears not to have been incorporated into the curricula of nursing schools in Europe (21).

The addition of CAM elements in biomedicine education is gaining popularity globally, and is becoming a trend in medical education reform in several developed countries such as USA, UK, Canada, Australia, and Germany (24). Is there also a need for CAM education in future nursing programs? What is the overall knowledge level of and attitude toward CAM among NSs? To the best of our knowledge, there is no definitive evidence available that addresses these questions. We conducted this systematic review aiming to understand NSs' overall rated knowledge of, attitude/belief toward, and practice/previous personal experience of usage (KAP) toward CAM in surveys, which is also expected to develop a new, more substantial interpretation of this research theme.

## Materials and methods

### Registration

The methods adopted for current systematic review are consistent with the guidelines detailed on *Preferred Reporting Items for Systematic Reviews and Meta-Analyses (PRISMA) 2020 Statement* checklist (25). The protocol for this systematic review has been registered in the Prospective Register of Systematic Reviews (PROSPERO): No. CRD42022300602.

### Eligibility criteria

Only formally published cross-sectional quantitative studies investigating the primary outcome of KAP toward CAM therapies by NSs were included in this review. Studies were also determined for NSs' major sources of CAM information, barriers to the use of CAM as perceived by NSs, and/or NSs' inclination to recommend CAM therapies to patients, which were viewed as the extension of KAP concept. Regardless of age, gender, race, grade, and academic level (diploma, undergraduate, or postgraduate), students majoring in nursing (excluding majoring in midwifery) could be included. Studies involving multiple professional groups [e.g., NSs versus (vs.) students with other healthcare background, or NSs vs. nursing faculty, etc.] were also included if they presented data separately for NSs. NSs' KAP toward CAM could be macroscopic or focused on a specific health condition (e.g., NSs' KAP toward CAM in stress relief or in the care of cancer survivors, etc.).

The following exclusion criteria were established. Intervention studies, reviews, case reports, study protocols, and letter to editors were excluded. Qualitative studies were also excluded as the current review aims to understand the overall KAP levels of CAM among NSs rather than the underlying causality. In this review, we considered CAM therapies as a broad umbrella term rather than focusing on specific products or practices. Therefore, studies focusing on NSs' KAP of a particular CAM therapy were excluded. Consistently, we also did not use a specific type of CAM therapy as a term for retrieval.

### Search terms and search strategy

Following consultation with a professional health sciences librarian with a nursing background who assisted in development of the overall search strategy, we used filters to reliably identify relevant studies published in English or Chinese, and undertook a comprehensive search of four English electronic databases and four Chinese electronic databases—MEDLINE (*via* PubMed), EMBASE (*via* OVID), Nursing & Allied Health Database (ProQuest), AMED: Allied and Complementary Medicine Database, China biomedical literature service system (SinoMed), China National Knowledge Infrastructure (CNKI), Chongqing VIP database (CQVIP), and Wanfang database—from their inception through to 28 April 2022. The search was carried out by combining search terms from three categories: (1) NS, (2) CAM, and (3) KAP (Search terms were listed in Appendix 2). Only studies that were formally published and available as full-text paper were included. To ensure literature saturation, searches were also complemented by an extensive manual retrieval of any additional articles meeting eligibility criteria that were cited in the reference lists of the included papers and relevant reviews, to avoid potential omission (see Appendix 3 for detailed search strategies used in search of the literature).

### Selection of studies and data extraction

Two researchers independently screened the titles and abstracts for eligibility. The full-text was then acquired and cross-checked for eligibility (FY-Z and WJ-Z). Two standardized and predetermined data forms were employed to extract the following information from each study: identification/demographic information (first author, year of publication, and country), study design [application of CAM in general or in targeted disease/health condition/clinical setting, sampling method, study characteristics (sample size investigated/sample size completed/response rate/context), participants characteristics (age range and average age, and gender ratio), control group characteristics (if available), outcome measure/instrumentation and its reliability and validity], and major findings and conclusions (results of KAP, main source of CAM information, the importance of different types of evidence for NSs' acceptance/use/recommendation of CAM, and other additional key results). In each study, information regarding the top three CAM therapies best known to NSs, the top three CAM therapies considered by NSs to be most effective, and the top three CAM therapies that NSs are most likely to recommend to patients were also collected and extracted.

### Study quality and risk of bias assessment

Two assessors (FY-Z and QQ-F) performed standalone appraisal of methodological quality and risk of bias of each study using the 11-item *Agency for Healthcare Research and Quality (AHRQ)* checklist which was recommended for cross-sectional studies (26). Each item was answered with “YES,” “NO,” or “UNCLEAR,” answering “yes” to score 1 point, and 0 point for the rest. Quality of each study was then rated as low quality (0–3 points), moderate quality (4–7 points), or high quality (8–11 points) (26). Any discrepancies and/or conflicts were resolved through consulting with a third assessor (WJ-Z).

### Data synthesis

All measurement instruments employed in the eligible studies were self-developed questionnaires/scales. Hence, the data derived were too heterogeneous to be statistical pooled for quantitative analysis. Furthermore, the modalities/types of CAM therapy investigated in different studies varied substantially. Given these issues, only a thematic summary, in a narrative fashion, of the results and findings was provided. The relevant information from the included studies was hence extracted and described in categories.

## Results analysis

### Study selection

**Figure 1:**
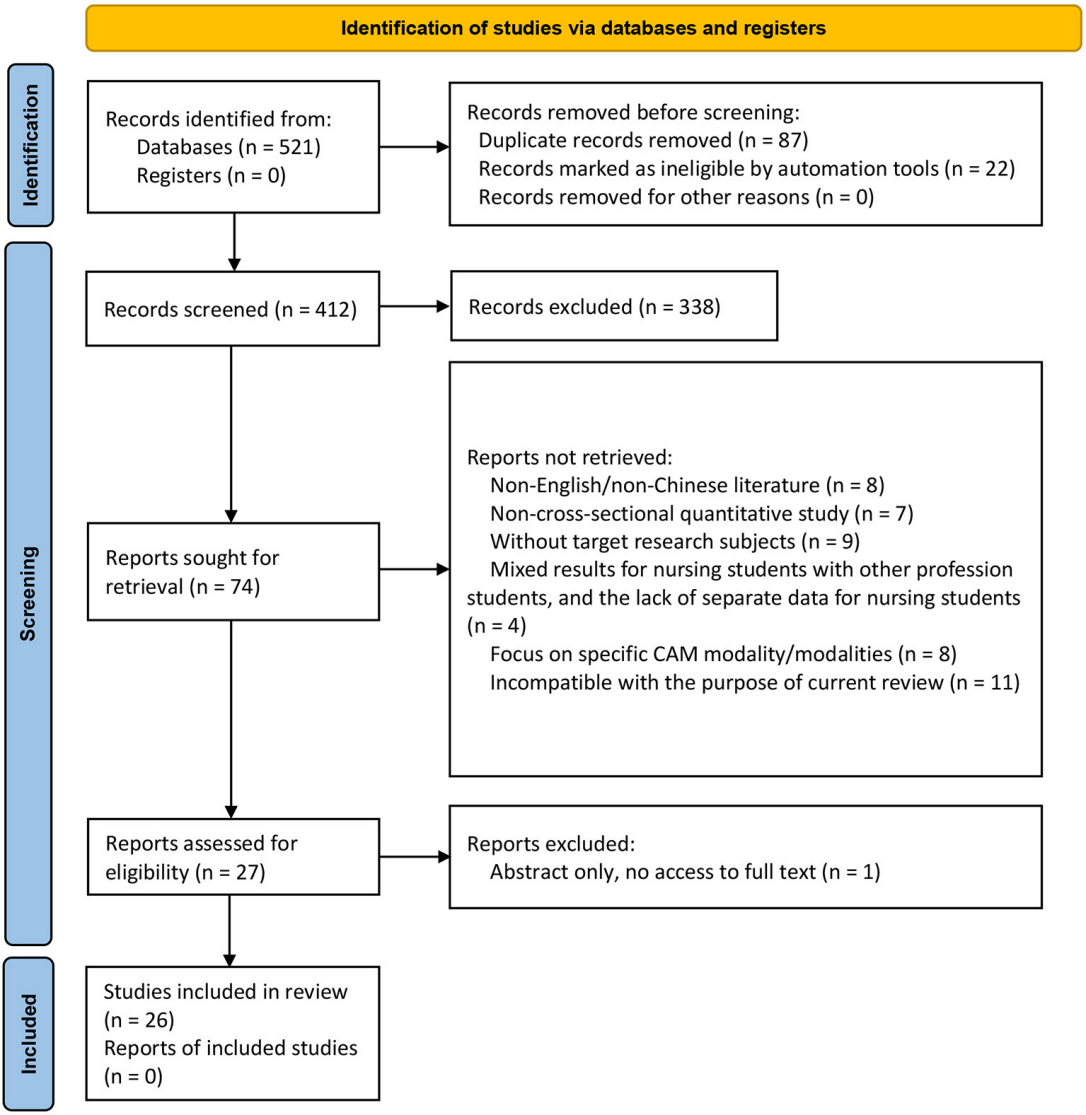
Flow diagram of the study selection process.

A total of 521 articles were identified using our search strategy in the initial search. After removal of the duplicates and articles with unrelated titles/abstract in the preliminary screening process, followed by a careful full-text screening, 26 studies eventually met the predefined criteria (Figure 1). All included studies were published in English. We did not identify any eligible papers available from any Chinese database. The discarded studies with detailed justifications for their exclusion are illustrated in Appendix 4.

### Study characteristics

Of the 26 included cross-sectional studies, most studies provided complete data on demographic characteristics (e.g., gender, age, etc.), with participants representing 12 different countries. Eleven studies were from Turkey (8, 27–36), four were from US (15, 16, 37, 38), two were from Australia (14, 39), with one from each of the following countries: UK (40), Canada (41), Norway (42), Hungary (43), Korea (44), Malaysia (45), India (46), South Africa (13), Sierra Leone (9). The convenience sampling method was adopted for all studies. Twenty-three studies investigated NSs' KAP toward CAM in general, while the remaining three studies (30, 33, 40) investigated NSs' KAP toward the application of CAM amongst cancer survivors and/or palliative care settings (Table 1).

**Table 1:** Study characteristics and outcome measurement tools of 26 included studies.

**References**	**Country**	**Target disease/condition**	**NSs**					**Control population**	**Outcome measures for NSs' KAP level**	
			**Sample size [sample size investigated/sample size responded/response rate (%)]**	**Context**	**Age (average)**	**Male/female**	**Sampling method**	**Population [sample size investigated/sample size responded/response rate (%)/context (▴represents that the setting context is the same as that of NSs)]**	**Instrumentation**	**Reliability**
Avino (15)	USA	Not limited	1,420/578/40.7%	Eight schools of nursing in USA	18–55 (27)	55/523	Convenience	Nursing faculty (155/117/75.5%/▴)	23-item questionnaire consisted of nine sections: 1. General attitudes and barriers to the use of CAM (scoring: very strongly agree to very strongly disagree) 2. Primary worldview guiding personal health view 3. Spiritual or religious beliefs influencing attitudes (scoring: yes; yes, somewhat; no; or no opinion) 4. Sources of CAM information 5. Evidence for use in CAM practice (scoring: essential to unimportant) 6. Effectiveness of CAM modalities (scoring: highly effective to harmful; or no opinion) 7. Previous education/training and further CAM training needs 8. Personal use of CAM (scoring: no, would not consider using it; no, but would consider using it; yes, have used it with positive outcomes; yes, have used it with neutral outcomes; yes, have used it with negative outcomes) 9. CAM approaches in practice (scoring: would not recommend; would endorse, but not personally provide or refer; would personally provide; would refer to a CAM practitioner; unsure)	NR
Baugniet et al. (41)	Canada	Not limited	NR/86/NR	Two universities in Canada	25–29 (NR)	NR/NR	Convenience	Students of PT (NR/90/NR/▴), OT (NR/101/NR /▴), pharmacy (NR/102/NR/▴), and medicine (NR/61/NR/▴)	A questionnaire focused on the NSs' perceptions of the general field of CAM	NR
Cinar et al. (27)	Turkey	Not limited	152/152/100%	One health school (university) in Turkey	17–24 (20.09 ± 1.6)	23/129	Convenience	N/A	1.17-item “Personal Information Form” 2. HCAMQ	Cronbach's alpha 0.72
Halcón et al. (16)	USA	Not limited	174/120/70.0%	One university in USA	21–57 (NR)	10/110	Convenience	Nursing faculty (76/50/65.7%/▴)	Questionnaire consisted of four sections: 1. Demographic information 2. Overall attitudes toward cam 3. Attitudes and information about training, personal use, perceived barriers, intent to integrate CAM into clinical practice 4. Primary worldviews or frameworks guiding personal health views, and influence of own spiritual or religious beliefs on attitudes toward CAM	NR
Hassan et al. (45)	Malaysia	Not limited	NR/33/NR	One university in Malaysia	NR (NR)	NR/NR	Convenience	Medical students (NR/41/NR/▴)	Questionnaire consists of three sections: 1. Knowledge of CAM 2. Attitudes toward CAM 3. Practice of CAM	Cronbach's alpha 0.878
James et al. (9)	Sierra Leone	Not limited	10/9/90.0%	One university in Sierra Leone	≥ 21 (NR)	2/7	Convenience	Medical (46/44/95.7%/▴) and pharmacy students (11/11/100%/▴)	CHBQ	NR
Kavurmaci et al. (28)	Turkey	Not limited	655/359/54.8%	One university in Turkey	NR (NR)	NR/NR	Convenience	Midwifery (195/170/87.2/▴) and dietetics students (169/142/84.0%/▴)	29-item questionnaire consists of three sections: 1. Demographic features 2. Knowledge about cam (scoring: yes; no; or do not know); from which source respondents obtained attitudes and opinions; use of cam 3. Whether CAM should be integrated into the education programs	NR
Kim et al. (44)	Korea	Not limited	400/98/24.5%	One school of nursing in Korea	NR (NR)	15/83	Convenience	Nursing faculty (55/100/55.0/▴)	Questionnaire A: demographic characteristics questionnaire B: knowledge of and experience with CAM [9 yes-or-no knowledge/experience items and 1 frequency item (scoring: never to very often)] questionnaire C: 10-item attitude state tool (scoring: 1= strongly disagree; 5 = strongly agree)	Cronbach's alpha 0.886 (attitude state tool)
Kreitzer et al. (37)	USA	Not limited	NR/NR/>50%	One university in USA	NR (NR)	NR/NR	Convenience	1. Nursing faculty (NR/50/>50%/▴) 2. students (NR/NR/>50%/▴) and faculty (70/31/44%/▴) of pharmacy 3. students (NR/NR/>50%/▴) and faculty (NR/64/>50%/▴) of medicine	Questionnaire consists of three sections: 1. socio-demographic characteristics 2. Overall attitudes toward cam 3. Attitudes and information about training, personal use, perceived barriers, the intent to integrate CAM into clinical practice (scoring: 1 = very strong agreement; 6 = very strong disagreement)	NR
Laurenson et al. (40)	UK	Cancer and palliative care	51/51/100%	One university in UK	≥18 (NR)	3/48	Convenience	N/A	A “closed question” questionnaire assessing NSs' knowledge of and attitudes toward CAM therapies in cancer and palliative care	NR
Özşaker (29)	Turkey	Not limited	224/224/100%	One university in Turkey	20–32 (22.87 ± 1.37)	31/193	Convenience	N/A	Questionnaire A: Student Identification Form (1. socio-demographic characteristics; 2. questions regarding whether NSs are familiar with CAM modalities; what do NSs think about the effectiveness of these modalities; whether NSs would like to be trained in these modalities) questionnaire B: HCAMQ	Cronbach's alpha 0.74 (HCAMQ)
Oztekin et al. (30)	Turkey	Cancer patients	1,057/640/60.5%	Six schools of nursing at five universities in Turkey	18–34 (21.5 ± 1.7)	0/640	Convenience	N/A	Questionnaire consists of three sections: 1. Demographic information and basic information on CAM 2. Four questions for each CAM modality (scoring: 0 = no, I have not; 1 = yes, I have)	NR
Pettersen et al. (42)	Norway	Not limited	3,170/317/10%	16 Norwegian colleges of nursing	NR (NR)	44/273	Non-probability convenience	Students of PT (300/63/21%/three Norwegian colleges of PT), social educator (590/59/10%/five Norwegian colleges of social educator), and radiography (126/34/27%/two Norwegian colleges of radiography education)	254-item questionnaire consists of three sections: 1. Demographic characteristics 2. “scientific evaluation-test” of health news briefs 3. Several statements involving positive attitude toward CAM, paranormal beliefs, requests, anti-scientistic, religious, spiritual, negative attitude toward science and scientists, and willingness to include CAM in education (scoring: 1 = strongly negative; 5 = strongly positive)	NR
Pirincci et al. (31)	Turkey	Not limited	550/489/88.9%	One university in Turkey	NR (21.29 ± 2.02)	214/275	Convenience	N/A	23-item questionnaire consists of two sections: 1. Socio-demographic characteristics 2. Use, knowledge, view of cam, and source of cam information	NR
Poreddi et al. (46)	India	Not limited	143/122/85.3%	One university in India	NR (20.6)	0/122	Non-probability convenience	N/A	Questionnaire consists of four sections: 1. Five-item demographic 2. 11-item perceived effectiveness/knowledge of CAM 3. General attitudes/perceptions of CAM 4. Perceived barriers to use of CAM	NR
Reuter et al. (38)	USA	Not limited	1. Pre-NSs (328/326/99.4%) 2. NSs (104/104/100%)	One university in US	1. Pre-NSs [18–30 (18.9 ± 1.2)] 2. NSs [20–51 (22.7 ± 3.8)]	1. Pre-NSs (32/294) 2. NSs (10/94)	convenience	N/A	Online survey ”*Students' Knowledge of and Attitude toward Holistic Medicine Practices*“ for pre-nursing students; online survey ”*Health Professions Students' Knowledge of and Attitude toward Holistic Medicine Practices*“ for NSs	NR
Sahin et al. (32)	Turkey	Not limited	390/264/67.7%	One university in Turkey	18–26 (20. 6 ± 1.77)	72/192	Convenience	N/A	Questionnaire consists of two sections: 1. Socio-demographic characteristics 2. CAM knowledge	NR
Sárváry et al. (43)	Hungary	Not limited	124/83/66.9%	One university in Hungary	18–31 (NR)	NR/NR	Convenience	Students of midwifery (204/126/61.8%/▴) and health visitor (196/105/53.6%/▴)	Questionnaire consisted of six sections: 1. General attitudes [12 items including 7 positive statements and 5 negative statements (scoring: 1 = strongly disagree; 7= strongly agree)] 2. Knowledge of CAM modalities (scoring: none; very little; some; a lot) 3. Sources of CAM information 4. Personal use and perceived effectiveness of CAM [scoring: yes/no; 1 = completely ineffective; 7 = completely effective] 5. Integration of CAM modalities into undergraduate curricula and health care system (scoring: 1 = strongly disagree; 7 = strongly agree) 6. Evaluation of CAM course [scoring: yes/no for if the respondent has participate in the CAM course; how much of CAM course was useful (1 = completely useless; 7 = completely useful)]	Cronbach's alpha 0.851 and Guttman λ_2_ = 0.858 (12-item questionnaire for attitude)
Topuz et al. (33)	Turkey	Cancer patients	192/148/77.1%	One university in Turkey	NR (21.09 ± 1.54)	2/146	Convenience	N/A	A literature-based questionnaire consists of three sections: 1. Demographical characteristics and experiences of CAM practices 2. Knowledge of and attitudes toward CAM 3. Opinions regarding use of CAM in cancer patients	NR
Turker et al. (34)	Turkey	Not limited	354/323/91.2%	One military medical school in Turkey	NR (NR)	0/323	Convenience	N/A	Questionnaire consists of three sections: 1. Demographic characteristics 2. Multiple-choice questions related to frequently used cam therapies 3. Attitudes toward CAM	NR
Uzun et al. (35)	Turkey	Not limited	280/276/98.6%	One university in Turkey	17–25 (20.16 ± 1.45)	0/276	Convenience	N/A	Questionnaire consisted of three sections: 1. General questions (sources of CAM information; attitudes toward inclusion of CAM into nursing curricula/practices) 2. Knowledge (scoring: no idea; a little knowledge; enough knowledge); awareness of therapeutic effect of CAM therapy (scoring: harmful; no idea; beneficial); whether offer CAM therapy to patient (scoring: offer; undecided; do not offer) 3. Perceptions of use and effect of CAM (from strongly disagree to strongly agree)	Cronbach's alpha 0.79
van et al. (13)	South Africa	Not limited	202/119/58.9%	One university in South Africa	≥18 (NR)	29/90	Convenience	N/A	12-item questionnaire consists of five sections: 1. Demographic characteristics 2. Personal use (scoring: never; only when needed; monthly; weekly; daily) 3. Recommendations to patients and enquiring about CAM use from patients 4. Knowledge of CAM (scoring: none; some; a lot) 5. Attitude toward CAM [23 statements (scoring: 1 = strongly disagree; 5 = strongly agree)]	1. Cronbach's alpha 0.87 (knowledge) 2. Cronbach's alpha 0.75 (attitude) 3. Cronbach's alpha 0.67 (use)
Walker et al. (39)	Australia	Not limited	321/316/98.4%	One university in Australia	≥18 (27.7 ± 9.0)	21/295	Convenience	Chiropractic students (227/225/99.1%/▴)	Questionnaire A: 15-item questionnaire for knowledge assessment (scoring: good knowledge;, some knowledge; aware; unaware) questionnaire B: modified 10-item CHBQ for attitudes/beliefs assessment (scoring: 1 = absolutely disagree; 7 = absolutely agree) questionnaire C: 10-item questionnaire investigating factors that influenced attitude (scoring:1 = not at all influential; 7 = highly influential)	NR
Wilkinson et al. (14)	Australia	Not limited	NR/105/NR	One university in Australia	NR (NR)	NR/NR	Convenience	Students of pharmacy (NR/69/NR/▴) and biomedical science (NR/97/NR/▴)	Questionnaire consists of two sections: 1. Demographic characteristics 2. Use of CAM during the past 12 months (CAM practitioners visited, products used, main sources of information about CAM, attitudes to CAM and conditions suffered)	NR
Yildirim et al. (8)	Turkey	Not limited	589/477/81.0%	One university in Turkey	17–29 (21.76)	NR/NR	Convenience	Medical students (1,353/495/36.6%/▴)	Questionnaire consists of four sections: 1. Socio-demographic characteristics 2. General attitudes toward cam 3. Sources of cam information 4. Knowledge of CAM, opinion of CAM's clinical usefulness, willingness to recommend CAM, and personal use of CAM	NR
Yilmaz et al. (36)	Turkey	Not limited	NR/458/NR	One faculty of health sciences in Turkey	NR (NR)	NR/NR	Convenience	Students of nutrition and dietetics (NR/159/NR/▴), PT (NR/119/NR/▴), and rehabilitation	1. A students' description form 2. ACTS 3. PHNS	1. Cronbach's alpha 0.84 (ACTS) 2. Cronbach's alpha 0.78 (PHNS)

NR, no records; N/A, not applicable; NSs, nursing students; PT, physiotherapy; OT, occupational therapy; CAM, complementary and alternative medicine; CHBQ, CAM Health Belief Questionnaire; PHNS, Perception of Health News Scale; ACTS, Attitude toward Using Complementary Treatments Scale; HCAMQ, Holistic Complementary and Alternative Medicine Questionnaire.

▴ represents that the setting for control population is the same as that of NSs.

Of all the included studies, 12 studies (13, 27, 29–35, 38, 40, 46) investigated KAP toward CAM among NSs only; while the remaining studies further compared the differences in KAP toward CAM between NSs and other groups/cohort, such as nursing faculty, or students/faculty of other health professions (e.g., medicine, pharmacy, rehabilitation/physiotherapy/occupational therapy, midwifery, radiography, nutrition/dietetics, chiropractic, biomedical science, social educator, or health visitor, etc.; (Table 1)).

All except one study (37) described the total number of NSs who participated in the studies, ranging from 9 to 640 individuals. The participants' degrees ranged from pre-nursing to postgraduate majoring in nursing. They therefore span a wide range of ages. Eighteen studies described the gender ratio of participated NSs, with the majority of participants being female NSs. All except four studies (14, 36, 41, 45) reported questionnaire/scale response rates amongst the participants, ranging from 10 to 100% (Table 1).

All except two studies (9, 36) employed or at least included one self-administrated questionnaire/scale as the primary outcome measure. These questionnaires/scales, in general, were developed by the researchers on the basis of expert opinion, review of textbooks, or other authoritative sources. Dimensions assessed by the questionnaires/scales differed from study-to-study, while typically comprised at least two sections: (1) items for collecting socio-demographic data; and (2) items for collecting NSs' level of knowledge of, and/or attitude/belief toward, and/or personal usage experience of each common CAM therapy. Some questionnaires/scales also covered items associated with (1) primary sources of CAM information; (2) perceptions of the effectiveness of each CAM modality; (3) willingness to recommend CAM therapies to patients, or willingness to provide patients with referrals to CAM services; (4) inclination to further CAM learning, or perceptions to incorporate CAM into conventional nursing education; and/or (5) barriers to using or integrating CAM. Measuring tools in five studies were adapted from previously developed questionnaires/scales, including CAM Health Belief Questionnaire (CHBQ) (9, 39), Holistic Complementary and Alternative Medicine Questionnaire (HCAMQ) (27, 29), or Attitude toward Using Complementary Treatments Scale (ACTS) (36). Unfortunately, these three tools were not developed specifically for NSs. It is also slightly unfortunate that psychometric properties such as Cronbach's coefficient alpha of the developed questionnaires/scales were reported in only eight (13, 27, 29, 35, 36, 43–45) of the 26 studies. One of the eight studies also tested the Guttman λ_2_ of the questionnaire (43). Those tests are conducted to assess the reliability of the questionnaires/scales included (Table 1).

### Study quality appraisal

Of the 26 studies included, one (41) (3.8%) was rated as low-quality study, eight (30.8%) (8, 9, 13, 27, 36, 38, 39, 43) were rated as high-quality studies, and the remaining 17 (65.4%) were rated as moderate-quality studies. Due to the lack of description of the assessment and/or control methods of confounding factors and the lack of follow-up data, all studies were appraised as high risk of bias in item 8 and item 11 of the checklist developed by AHRQ, respectively (Table 2).

**Table 2:** Methodological quality assessment of 26 included studies based on AHRQ checklist.

**Studies\items**	**Item 1**	**Item 2**	**Item 3**	**Item 4**	**Item 5**	**Item 6**	**Item 7**	**Item 8**	**Item 9**	**Item 10**	**Item 11**	**Total scores out of 11**	**Level of quality**
Avino (15)	Y	Y	N	Y	U	N	N	N	N	Y	N	4	Moderate
Baugniet et al. (41)	Y	Y	N	U	U	N	N	N	N	Y	N	3	Low
Cinar et al. (27)	Y	Y	Y	Y	U	Y	Y	N	Y	Y	N	8	High
Halcón et al. (16)	Y	N	Y	U	U	N	Y	N	Y	Y	N	5	Moderate
Hassan et al. (45)	Y	Y	Y	U	U	Y	N	N	N	N	N	4	Moderate
James et al. (9)	Y	Y	Y	Y	Y	N	Y	N	Y	Y	N	8	High
Kavurmaci et al. (28)	Y	N	Y	U	U	N	Y	N	Y	Y	N	5	Moderate
Kim et al. (44)	Y	N	Y	U	Y	Y	Y	N	Y	Y	N	7	Moderate
Kreitzer et al. (37)	Y	N	N	U	U	N	Y	N	Y	Y	N	4	Moderate
Laurenson et al. (40)	Y	N	N	N	Y	N	Y	N	Y	Y	N	5	Moderate
Özşaker (29)	Y	N	Y	U	U	Y	Y	N	Y	Y	N	6	Moderate
Oztekin et al. (30)	Y	Y	N	U	Y	N	Y	N	Y	Y	N	6	Moderate
Pettersen et al. (42)	Y	N	N	U	U	N	Y	N	Y	Y	N	4	Moderate
Pirincci et al. (31)	Y	Y	Y	N	U	N	Y	N	Y	Y	N	6	Moderate
Poreddi et al. (46)	Y	Y	Y	U	U	N	Y	N	Y	Y	N	6	Moderate
Reuter et al. (38)	Y	Y	Y	Y	Y	N	Y	N	Y	Y	N	8	High
Sahin et al. (32)	Y	U	N	U	U	N	Y	N	Y	Y	N	4	Moderate
Sárváry et al. (43)	Y	Y	Y	Y	U	Y	Y	N	Y	Y	N	8	High
Topuz et al. (33)	Y	N	U	U	U	N	Y	N	Y	Y	N	4	Moderate
Turker et al. (34)	Y	N	Y	U	U	N	Y	N	Y	Y	N	5	Moderate
Uzun et al. (35)	Y	Y	N	Y	U	Y	Y	N	Y	Y	N	7	Moderate
van et al. (13)	Y	Y	N	Y	Y	Y	Y	N	Y	Y	N	8	High
Walker et al. (39)	Y	Y	Y	Y	Y	N	Y	N	Y	Y	N	8	High
Wilkinson et al. (14)	Y	Y	Y	Y	Y	N	Y	N	Y	U	N	7	Moderate
Yildirim et al. (8)	Y	Y	Y	Y	Y	N	Y	N	Y	Y	N	8	High
Yilmaz et al. (36)	Y	Y	Y	Y	U	Y	Y	N	Y	Y	N	8	High

Y, yes; N, no; U, unclear; AHRQ, Agency for Healthcare Research and Quality.

Item 1: Define the source of information (survey, record review); Item 2: List inclusion and exclusion criteria for exposed and unexposed subjects (cases and controls) or refer to previous publications; Item 3: Indicate time period used for identifying patients; Item 4: Indicate whether or not subjects were consecutive if not population-based; Item 5: Indicate if evaluators of subjective components of study were masked to other aspects of the status of the participants; Item 6: Describe any assessments undertaken for quality assurance purposes (e.g., test/retest of primary outcome measurements); Item 7: Explain any patient exclusions from analysis; Item 8: Describe how confounding was assessed and/or controlled; Item 9: If applicable, explain how missing data were handled in the analysis; Item 10: Summarize patient response rates and completeness of data collection; Item 11: Clarify what follow-up, if any, was expected and the percentage of patients for which incomplete data or follow-up was obtained. 1 point for “Yes,” and 0 point for the rest. 0–3 points for “low quality,” 4–7 points for “moderate quality,” and 8–11 points for “high quality.”

### KAP toward CAM

#### Knowledge level of CAM

Knowledge level of CAM was generally measured by asking whether NSs were familiar with or had heard about each CAM modality. Respondents usually provided “yes-or-no” and/or frequency (e.g., never to very often, no knowledge to know very well, no idea to sufficient, etc.) as answers. Fourteen studies (8, 9, 13, 29, 31–35, 38–40, 43, 44) evaluated the NSs' overall level of knowledge and the findings were consistent, that is, although NSs' knowledge level differed/varied depending on the specific CAM modality, the overall level was limited, less than satisfactory, and the surveyed students felt poorly informed (Table 3).

**Table 3:** NSs' knowledge, attitude, use, barriers to use, recommendation, and source of information toward CAM therapies.

**References**	**Major results regarding NSs' KAP toward CAM [Primary outcomes] and comparison with control population (% represents the proportion of NSs respond)**							**Other results related to NSs' KAP toward CAM [Secondary outcomes]**			
	**NSs' KAP toward CAM**						**vs. control population (if available)**				
	**NSs' knowledge about CAM**		**NSs' attitude** **toward CAM**			**NSs' practice of CAM**					
	**Overall**	**CAM modalities NSs most expect to learn**	**Overall**	**Key factors that influence attitude**	**Agreement that CAM is a threat to public health**	**NSs' personal use of CAM (overall use/primary reason/top used modality/major barrier to use)**		**NSs' source of CAM information**	**CAM modalities most likely to be recommended to patients by NSs**	**Importance of different types of evidence for accepting/using/recommending CAM treatment among NSs**	**Additional key and valuable findings**
Avino (15)	NR	1. Massage (12%) 2. Nutritional supplements (11%) 3. Therapeutic touch/healing touch (10%)	– Positive – 81% NSs want further training that is sufficient to advise patients about CAM use or to personally provide CAM therapies – 81% NSs express that CAM practice should be included in the curricula – 88% NSs agree that knowledge about CAM is important to their future career	Spiritual and religious beliefs (reported by 66% NSs)	5%	NR/NR/NR/lack of evidence (96%) and lack of staff training (96%)	1. Nursing faculty and NSs show similar attitudes toward CAM in all areas 2. More NSs (81%) than nursing faculty (62%) want further training	1. Peer professionals (73%) 2. Mass media (55%) 3. Internet (53%)	NR	NR	1.50% NSs use western biomedicine to guide personal health view and 46% NSs use a combination of western biomedicine and another health tradition to guide health view 2. 82% NSs will consider using all CAM therapies personally, and 68% NSs will use CAM in clinical care by endorsing, providing personally, or referring patients to a CAM practitioner 3. 93% NSs report that evidence is essential or somewhat essential/important when recommending CAM therapies to patients
Baugniet et al. (41)	NR	NR	– Positive – More than 66% NSs have interests in receiving training to practice a form of CAM – 70.9% NSs support that CAM should be taught as a separate course in their curricula	1. Levels of exposure and training 2. Culture and values internalized during the students' training	0	44.7%/NR/ massage/ NR	NSs are more likely to consult a CAM practitioner than other healthcare students	NR	NR	1. Success in practice (96.4%) 2. Patient reports (83.1%) 3. RCTs involving humans (72.3%)	Healthcare students generally have more knowledge about those CAM therapies considered to be the most mainstream (e.g., chiropractic, massage therapy, acupuncture, etc.) and less knowledge about those less-widely accepted CAM therapies (e.g., homeopathy, faith healing, and reflexology, etc.)
Cinar et al. (27)	NR	NR	– Positive – 61.3–64.5% NSs express that there should be a course on CAM in the curricula – 57.8–62.3% NSs support CAM should be used in patient care practice	NR	NR	NR/NR/NR/NR	N/A	NR	NR	NR	50.7% NSs state that they have no information on CAM
Halcón et al. (16)	NR	NR	– Positive – 93.3% NSs support the integration of CAM into curricula and agree that information on CAM is significant to their professional clinical practice – More than 33.3% NSs desire sufficient training to be able to advise patients about the use of CAM therapies	NR	2.5%	98.3%/NR/ massage/lack of evidence	NSs (95%) are more likely than nursing faculty (94%) to hope to have some CAM therapies available to patients in their practice or networks	1. Peer professionals (82.5%) 2. Professional journals (78.3%) 3. Other health care providers (65.9%)	NR	1. Success in practice (79.2%) 2. Patient reports (74.2%) 3. Published case studies (74.2%)	NSs are more interested in improving their knowledge for providing informed referrals than in gaining education aimed at incorporating CAM therapies into their own practice
Hassan et al. (45)	NR	NR	– Positive – Support the integration of CAM into curricula and agree that information on CAM is significant to them as future nurse	Level of knowledge	Some NSs	NR/NR/NR/NR	NSs are more knowledgeable and positive about CAM, and more willing to practice CAM than medical students	NR	NR	NR	The more CAM knowledge NSs have, the more positive they will practice in their clinical settings
James et al. (9)	33.3% NSs know CAM	NR	– Positive – 77.8% NSs have interests in studying CAM – 71.4% NSs prefer CAM to be an elective module rather than a compulsory module, while 28.6% NSs make an opposite decision	NR	0	55.6%/NR/ herbal/NR	1. there is no significant difference among the three groups with respect to self-reported use, recommendation of CAM, and perceived knowledge of CAM 2. NSs are less positive about CAM than medical students; no significant difference in attitude toward CAM between NSs and pharmacy students	1. Books/journals (55.6%) 2. CAM practitioner (22.2%) 3. Media (11.1%)	1. Herbal 2. Spiritual/prayer	NR	55.6% NSs indicate that they will recommend CAM to their patients
Kavurmaci et al. (28)	NR	NR	– Positive – Support the integration of CAM into curricula and agree that information on CAM is significant to them as future nurse	NR	NR	59.9%/NR/NR/NR	1. NSs' use of CAM is more than that of midwifery students (31.8%) and dietetics students (32.4%) 2. NSs are more favorable toward CAM use than midwifery students and dietetics students	NR	NR	NR	NR
Kim et al. (44)	Limited	NR	– Positive – Support the integration of CAM into curricula and support the content on CAM needs to be included in nursing licensure examinations	NR	NR	NR/NR/mineral and herbal therapy/NR	There is significant difference in knowledge level between NSs and nursing faculty (nursing faculty have more knowledge of meditation and acupuncture than that of NSs; NR for overall knowledge level or knowledge level for other CAM therapies)	NR	NR	NR	1. NSs indicate that CAM has its role in the community (83%) rather than in the hospital (75.8%) 2. NSs note that the use of CAM is more appropriate for advance practice nurses
Kreitzer et al. (37)	NR	NR	– Positive – NSs desire more training in CAM modalities, and have favorable attitudes toward the integration of CAM into education and clinical care	NR	5.3%	NR/NR/NR/lack of evidence	1. NSs are less positive about CAM than nursing faculty; more NSs than nursing faculty agree that CAM is a threat to public health 2. NSs are more positive about CAM than medical or pharmacy students; NSs are more likely than other healthcare students to use or consider using CAM therapies	NR	NR	NR	NSs have limited training of CAM
Laurenson et al. (40)	Limited	NR	– Positive – Need CAM education as a core module rather than an optional module	NR	21.6% NSs agree that CAM conflict with conventional medicine	NR/NR/NR/NR	N/A	NR	NR	NR	1. NSs require more information about CAM if they are going to help patients make informed choices 2. NSs are unsure if CAM may conflict with conventional treatment, but disagree that CAM are ineffective
Özşaker (29)	Limited	1. Music therapy (83.5%) 2. Phytotherapy (79.9%) 3. Acupuncture (78.6%)	– Positive and moderate – 73.2% NSs support that CAM can be applied in addition to conventional medical treatment – Most NSs want to incorporate CAM lessons into the curricula or learn more about CAM	NR	5.4%	NR/relaxation/music therapy/NR	N/A	1. Internet (68.8%) 2. Scientific/medical books (50%) 3. Family/relatives (48.2%)	NR	NR	Expectations and feedbacks of NSs should be taken into consideration in determining the education standards
Oztekin et al. (30)	NR	1. Nutritional therapy (89.5%) 2. Breathing therapies (87.2%) 3. Massage and manipulation/Tui-Na (87%)	– Positive – Support the integration of CAM into nursing curricula	NR	NR	NR/NR/music therapy/NR	N/A	NR	NR	NR	1. NSs intend to use CAM although they do not have formal education during the undergraduate program 2. NSs' personal use of CAM are limited 3. NSs have limited sources of CAM information
Pettersen et al. (42)	NR	NR	– Positive – Support the integration of CAM into health science education	1. Paranormal beliefs 2. Willingness to include CAM in education 3. Perception of whether reliable knowledge can be obtained by intuition/spiritual 4. Religious	NR	NR/NR/NR/NR	NSs have more positive attitude toward CAM and higher degree of willingness to include CAM in education than PT students have	NR	NR	NR	Discussing the scientific basis of claims of CAM effects is probably an ideal teaching approach
Pirincci et al. (31)	Limited (only 21.9% NSs feel they have enough knowledge on CAM)	NR	– Positive – 55.4% NSs agree that CAM methods and practice should be combined with conventional nursing practice – 56.2% NSs support that CAM practice should be included in nursing training programs	NR	21.5%	51.3%/treat health problems/herbal products/NR	N/A	1. Internet (68.7%) 2. Family (64.8%) 3. Friend (51.1%)	NR	NR	1.52.6% NSs would recommend CAM methods to others 2. The use of CAM therapies significantly increase with increases in NSs' ages and their parents' education status
Poreddi et al. (46)	NR	NR	– Positive – 93.0% NSs are in favor of including CAM in the nursing curricula – 76.2% NSs agree that CAM (including Ayurveda) knowledge is needed for future professionalism	NR	NR	NR/NR/NR/lack of evidence	N/A	NR	NR	NR	85.2% NSs agree that patients have the right to choose between modern medicine and CAM
Reuter et al. (38)	Limited	Pre-NSs: 1. Cell therapy (37.2%) 2. Acupuncture (31.4%) 3. Diet-based therapy (29.4%) NSs: 1. Aromatherapy (71.9%) 2. Music therapy (70.3%) 3. Meditation (64.1%)	– Positive (NSs are more positive than Pre-NSs) – Overall are interested in CAM (NSs show more interests than Pre-NSs do) – Expect to learn more about CAM in nursing programs, and plan on making CAM practices part of their future careers	Knowledge of or experience with CAM practices [learning about CAM practices may influence how NSs (including Pre-NSs) rate their attitude toward specific practice beyond personal experience]	NR	1. Pre-NSs (73%/NR/diet-based therapy/NR) 2. NSs (81.7%/NR/music therapy/NR)	N/A	NR	NR	NR	It should be noted that the importance of learning about a CAM practice for NSs' decision about which practices to make part of their career
Sahin et al. (32)	Limited (46.6% NSs do not know the definition of CAM)	NR	– Positive – 64% NSs support to have CAM included in nursing curricula [57.6% NSs prefer CAM to be an elective module rather than a compulsory module, while 6.4% NSs make an opposite decision] – 42% NSs support the use of CAM in future nursing practice	NR	NR	49.6/stress/NR/NR	N/A	1. Internet or TV (58.4%) 2. Newspaper, magazines, books (42.6%) 3. Family, relatives, or friends (35.9%)	NR	NR	1.66.3% NSs have not received any information about CAM 2. There is a positive correlation between CAM knowledge level and CAM recommendations 3. There is a positive correlation between the status of NSs benefitting from CAM and their recommendations of CAM
Sárváry et al. (43)	Limited	NR	– Positive – 59.8% NSs support the integration of evidence-based CAM therapies into curricula – 54.3% NSs support that CAM should be researched – 53.7% NSs support that the integration of CAM into health care will be effective – Most NSs prefer CAM to be an elective module (courses that are connected to their specialty) rather than a compulsory or an optional module (courses that are not absolutely related to their specialty)	NR	26.5% NSs agree that the increase in CAM use is dangerous because it raises unfounded hopes and leads to disappointment	NR/NR/ massage/NR	1. There is no significant differences in knowledge of and attitudes toward CAM between NSs, health visitor and midwifery students 2. The proportion of NSs participating in CAM courses is lower than that of midwifery students (61.5%) or that of health visitor students (84.7%)	1. Internet (83.1%) 2. Family members, relatives and friends (55.4%) 3. Scientific journals or books (51.8%)	NR	NR	1. There is no significant differences in the attitudes toward CAM between students who participate in and those who do not participate in the CAM course 2. Participants of CAM course prefer formal education as the source for obtaining CAM information
Topuz et al. (33)	Limited	NR	– Positive – 77% NSs support the integration of CAM into nursing curricula – 54.7% NSs support the integration of CAM into future nursing practice	Clinical experiences	26.4% NSs agree and 27% NSs slightly agree that CAM practices can affect medical treatment because of side effects	NR/NR/ NR/NR	N/A	1. Media (67.6%) 2. Internet (61.7%) 3. Books/journals (32.4%)	NR	NR	Lack of nursing faculty members and nurses training, and lack of evidence for practice are two major barriers to develop CAM curricula
Turker et al. (34)	Limited	NR	– Neutral – Most NSs support the integration of lectures of CAM practice into the curricula for the students who are enthusiastic about learning them	NR	40.6%	NR/NR/NR/NR	N/A	1. TV and radio (80.3%) 2. Internet (69.0%) 3. Newspapers/journals (48.6%)	NR	NR	62.9% NSs keep neutral for the statement “CAM therapies are effective and safe”
Uzun et al. (35)	Limited	NR	– Positive – 64.5% NSs support CAM to be integrated into the nursing curricula – 62.3% NSs support CAM therapies to be used in clinical practice	NR	49.6% NSs agree that CAM are not threat to public health	NR/NR/ NR/NR	N/A	1. Newspapers, magazines and TV (37.3%) 2. Nurse education program (26.1%) 3. Social sources, e.g., friends, and family environment in third place (25%)	1. Humor (56.1%) 2. Music therapy (53.2%) 3. Relaxation techniques (51.4%)	NR	1. NSs show willingness to offer CAM therapies if they have sufficient knowledge 2. Proper provision of medical data in CAM therapies will make it easier to apply and to decide which therapy will be used
van et al. (13)	48% NSs indicate very little CAM knowledge	NR	1. Neutral view (51.2%) 2. Positive/very positive (45.4%)	1. Philosophical congruency 2. Freedom of choice with regard to healthcare 3. Ease of access 4. Cost-effectiveness and perceived efficacy 5. Safety	12.6% NSs agree and 30.3% NSs strongly agree that CAM practice can affect medical treatment because of side-effects	44%/improve condition/ massage/ lack of knowledge	N/A	NR	1. Massage therapy (66%) 2. Homeopathy (53%) 3. Herbal medicine/ phytotherapy (42%)	NR	1.25.2 NSs agree and 22.7% NSs strongly agree that CAM is a branch of science 2. Almost half of NSs are willing to refer patients to a CAM practitioner 3. 45% NSs report that they always enquire about the CAM use from their patients
Walker et al. (39)	Limited	NR	Relatively positive	1. Scientific evidence 2. Personal experience 3. Cultural background	Some NSs	NR/NR/NR/NR	1. There is no significant difference in knowledge level between NSs and chiropractic students 2. Chiropractic students lean more favorably toward recommending CAM than NSs	NR	NR	NR	NR
Wilkinson et al. (14)	NR	NR	– Positive – The majority of NSs agree that CAM can improve quality of life	NR	NR	NR/stress/vitamin, minerals and other supplements/NR	1. There is few difference in attitudes toward CAM among different healthcare students 2. More pharmacy students (38.8%) and biomedical sciences students (35.4%) than NSs (19.6%) think CAM have only limited use	1. Friends 2. Family 3. Magazines	NR	NR	NR
Yildirim et al. (8)	Limited	NR	– Positive – 61.3% NSs support the integration of CAM into nursing curricula – 57.8% NSs support the integration of CAM into nursing care	NR	10.3%	NR/NR/prayer therapy/NR	1. NSs are more positive and knowledgeable than medical students about most of CAM modalities 2. NSs believe in CAM's clinical usefulness more than medical students 3. NSs use CAM individually more than medical students 4. NSs have more willingness than medical students about recommending CAM modalities to patients	1. Books (20.2%) 2. TV (18.8%) 3. Newspaper/journals (18.4%)	1. Relaxation techniques (80.5%) 2. Diet (80.4%) 3. Massage (79.0%)	NR	NR
Yilmaz et al. (36)	NR	NR	Positive	NSs' perception of health news may affect their attitudes toward using CAM therapies	NR	NR/considering harmless/NR/NR	There is no significant difference in attitudes toward CAM use between different healthcare students	NR	NR	NR	NSs have a low prevalence of CAM education

NR, no records; N/A, not applicable; NSs, nursing students; CAM, complementary and alternative medicine.

Fourteen studies compared nursing students with other healthcare students/faculty. In two studies (8, 45), NSs were found to be more knowledgeable than medical students about most CAM modalities. Three studies (9, 39, 43) reported no significant difference in knowledge levels of CAM between NSs and their counterparts [students with other healthcare backgrounds (e.g., medicine, pharmacy, midwifery, health visitor, and chiropractic, etc.) in same grade level]. No studies showed that NSs had lower knowledge levels of CAM than their counterparts. Whether the overall knowledge levels of CAM among NSs was higher or lower than that of nursing faculty was not disclosed in any of the included studies. Only one study reported that nursing faculty had more knowledge of meditation and acupuncture than NSs, but that study did not provide comparison results for other CAM modalities (44) (Table 3).

Four studies (15, 29, 30, 38) investigated which CAM modality NSs most desired further knowledge. In summary, NSs showed great interests in learning massage, nutritional/diet-based therapy, music therapy, acupuncture, meditation, aromatherapy, phytotherapy, and therapeutic touch/healing touch (Table 3).

#### Attitudes/beliefs toward CAM

All included studies outlined NSs' attitude or belief toward CAM. In only two studies (13, 34), more than half of the NSs expressed a neutral attitude toward CAM. In the remaining 24 studies, the majority of NSs (>50%) saw CAM positively. Specifically, most NSs respondents in these 24 studies supported the incorporation of CAM knowledge into existing nursing curricula or integration of CAM curricula into the conventional nursing education programs. Nevertheless, with respect to whether CAM courses should be delivered as elective or compulsory module, proponents in different studies expressed inconsistent views. In two studies (9, 32), most NSs showed preference for the CAM course to be taught as an elective module rather than a compulsory module, while few NSs expressed opposite inclination. In addition, as suggested in an aforementioned study (34) which respondents consisted of a majority of NSs who held neutral attitude about CAM, lectures on CAM practices should only be offered to NSs who are enthusiastic about CAM. However, respondents in another study argued for the opposite, by requesting CAM education integrated as a core rather than an optional module in the pre-registration curricula (40). In light of the responses from NSs, the author of one included study even advised education reformers or industry policy makers should consider incorporating CAM content into nursing licensure examination once CAM curricula is widely and fully integrated into nursing programs at all levels (44) (Table 3).

Eleven out of 26 studies examined students' desire for further CAM training, or their perceptions regarding the impact of CAM knowledge/information on their future career. In five of them, many NSs (33.3–81%) conveyed a desire to receive further or sufficient training so that be able to safely advise patients on the use of CAM (9, 15, 16, 37), or to practice a CAM modality (41). In eight studies, most NSs (54.7–93.3%) agreed that knowledge or information of CAM was important for their future careers (15, 16, 28, 32, 33, 38, 45, 46) (Table 3).

In seven studies, most NSs (55.4–73.2%) supported that CAM should be used in the patient care practice (8, 27, 35, 37), or agreed that CAM remedies can be applied in addition to conventional medical treatment (29, 43) and/or conventional nursing practices (31). In contrast, in another study, 21.6% of NSs considered CAM to be antithetical to/conflict with conventional medicine (40) (Table 3).

The question “*Do you agree that CAM is a threat to public health*” was asked in 12 studies, and the agreement with this question was less than half (0 to 40.6%) in each of these studies (8, 9, 15, 16, 29, 31, 34, 35, 37, 39, 41, 45). In two studies (13, 33), some NSs agreed (12.6–26.4%) or slightly agreed (27%) that CAM practices due to side-effects can affect conventional medical treatment. Another study (43) described that only 26.5% of NSs did not support increased utilization of CAM on the grounds that it would raises patients' unfounded hopes and lead to frustration (Table 3).

Nine studies further explored the potential factors influencing NSs' attitude toward CAM. In light of the results, level of knowledge, previous positive personal experience, and/or personal cultural background (including religious/beliefs) appeared to be the major factors (Table 3).

In most of the included studies (8, 28, 37, 42, 45), in comparison with students with other healthcare background, NSs generally showed a more positive attitude toward CAM (Table 3).

#### Practice/personal use of CAM

NSs' overall personal use of CAM therapies were depicted in eight studies (9, 13, 16, 28, 31, 32, 38, 41). The lowest rate of CAM use reported was 44% (13) and the highest rate of use at 98.3% (16). Reasons for CAM personal use were detailed in only six studies. The major reasons were reduction of stress or relaxation (14, 29, 32), treatment of general health problems (31), improvement of current health condition (13), and consideration of the harmlessness of CAM modalities (36). Twelve studies further detailed the most popular and widely used CAM by NSs, involving massage (13, 16, 41, 43), herbal medicine (9, 31, 44), music therapy (29, 38), diet-based therapy (including vitamin, minerals, and other supplements) (14, 38), and prayer therapy (8) (Table 3).

Five studies (15, 16, 37, 39, 46) examined the major barriers to CAM practice/personal use under the perception of NSs. Congruently, lack of evidence and lack of staff training were judged as the two most significant barriers (Table 3).

Seven studies did not display any information relevant to the practice/ personal use of CAM (Table 3).

As reported, in comparison with other healthcare professions students, NSs were more likely to consult a CAM practitioner (41), or use/consider using CAM therapies (8, 28, 37) (Table 3).

### CAM modalities best known to/most recognized by NSs

Of all the included studies, 15 determined NSs' knowledge/awareness rate of various CAM modalities (8, 9, 13, 29, 31–35, 38, 39, 41, 43–45). The top three modalities most familiar to NSs across each study are displayed in Table 4 (two studies counted only the two modalities most familiar to NSs). We noticed that massage, prayer/spirituality/spiritual healing, and herbal medicine were best known to NSs.

**Table 4:** Top three CAM modalities best known to nursing students in each study.

**References**	**NR**	**Acupuncture**	**Chiropractic**	**Aromatherapy**	**Diet/nutrition (including vitamins, minerals, and other supplements)**	**Herbal medicine**	**Music therapy**	**Massage**	**Meditation/imagery**	**Prayer/spirituality/spiritual healing**	**Relaxation techniques**	**Therapeutic touching**	**Yoga**	**Hypnosis**	**Exercise**	**Religious practices**
Avino (15)	•															
Baugniet et al. (41)			•					•				•				
Cinar et al. (27)	•															
Halcón et al. (16)	•															
Hassan et al. (45)								•		•	•					
James et al. (9)						•				•						
Kavurmaci et al. (28)	•															
Kim et al. (44)								•	•							
Kreitzer et al. (37)	•															
Laurenson et al. (40)	•															
Özşaker (29)		•					•							•		
Oztekin et al. (30)	•															
Pettersen et al. (42)	•															
Pirincci et al. (31)					•	•									•	
Poreddi et al. (46)	•															
Reuter et al. (38)		•							•				•			
Sahin et al. (32)									•	•					•	
Sárváry et al. (43)		•						•			•					
Topuz et al. (33)		•				•										•
Turker et al. (34)						•				•				•		
Uzun et al. (35)					•			•		•						
van et al. (13)				•		•		•								
Walker et al. (39)					•				•				•			
Wilkinson et al. (14)	•															
Yildirim et al. (8)								•	•	•						
Yilmaz et al. (36)	•															

NR, no records; The • symbol indicates the top three CAM modalities best known to nursing students in each study.

Seven studies (8, 16, 32, 34, 35, 41, 46) investigated the CAM modalities that NSs considered to be the most effective. The top three across each study were listed in Table 5. Therapies such as massage, herbal medicine, chiropractic, and diet/nutrition (including vitamins, minerals, and other supplements) were recognized by the nurses for their effectiveness.

**Table 5:** Top three CAM modalities considered to be the most effective by nursing students in each study.

**References**	**NR**	**Acupuncture**	**Chiropractic**	**Diet/ nutrition (including vitamins, minerals, and other supplements)**	**Herbal medicine**	**Homeopathy**	**Music therapy**	**Massage**	**Prayer/ spirituality/ spiritual healing**	**Relaxation techniques**	**Support groups**	**Ayurveda**
Avino et al. (15)	•											
Baugniet et al. (41)		•	•					•				
Cinar et al. (27)	•											
Halcón et al. (16)			•	•				•				
Hassan et al. (45)	•											
James et al. (9)	•											
Kavurmaci et al. (28)	•											
Kim et al. (44)	•											
Kreitzer et al. (37)	•											
Laurenson et al. (40)	•											
Özşaker (29)	•											
Oztekin et al. (30)	•											
Pettersen et al. (42)	•											
Pirincci et al. (31)	•											
Poreddi et al. (46)					•	•						•
Reuter et al. (38)	•											
Sahin et al. (32)				•	•			•				
Sárváry et al. (43)	•											
Topuz et al. (33)	•											
Turker et al. (34)					•			•	•			
Uzun et al. (35)							•	•			•	
van et al. (13)	•											
Walker et al. (39)	•											
Wilkinson et al. (14)	•											
Yildirim et al. (8)				•				•		•		
Yilmaz et al. (36)	•											

NR, no records; The • symbol indicates the top three CAM modalities considered to be the most effective by nursing students in each study.

By coincidence, massage and herbal medicine are the modalities both best known to NSs and ones the NSs perceive most effective (Tables 4, 5).

### CAM modalities that NSs tend to/are willing to recommend

Four studies (8, 9, 13, 35) investigated the CAM modalities that NSs inclined to or were willing to recommend to their patients in the future clinical practice. Massage, herbal medicine/phytotherapy, and relaxation techniques were cited by respondents in at least two of these four studies as one of the top three modalities that were preferred and could be recommended (Table 3).

### Importance of different types of evidence to CAM-related clinical decisions

Two studies (16, 41) rated the importance of different types of evidence for NSs in making clinical decisions with respect to CAM, such as acceptance, usage, or recommendation of CAM therapies. For a specific CAM modality, successful use in one's own practice and patient reports are judged by NSs as the most two important pieces of evidence, even more important than proven mechanism of action or proposed biological mechanism of action. Other crucial evidence included randomized controlled clinical trials (RCTs) involving humans and published case studies (16, 41). In both studies, RCTs involving animal studies were considered as the evidence with least importance (Table 3).

### Sources for accessing CAM information

Eleven studies (8, 9, 15, 16, 29, 31–35, 43) investigated the sources from which NSs acquire information regarding CAM. These sources of information mainly involve books, written-visual and mass media (newspapers/magazines/TV), internet, family/friends, peer professionals, health care providers, and education programs. For NSs, amongst those sources, the proportion of accessing CAM information from the media and the internet far exceeded the proportion of accessing CAM information from professional healthcare providers or scholastic education (Table 3).

## Discussion

### Summary of findings

It is clear that insufficient importance is given to CAM theory and modalities in contemporary formal nursing education curricula design. NSs' knowledge level of CAM, although overall limited and poorly informed, was generally comparable or higher than that of other health profession students within the same age/grade group. It is hypothesized that this may be related to acceptance by nurses of patient choice in self-care. The current level of CAM knowledge lags behind interest amongst NSs. Unfortunately, when there is insufficient knowledge gained in formal curricula, the media and internet are the primary sources for NSs to obtain CAM-related information or knowledge. The great interest and lack of credible source of information support an important need for change. Approximately 42.0–93.3% of NSs agreed that CAM ought to be combined with conventional medical/nursing practice to better serve patients' health and improve the quality of care available, or believed that information/knowledge on CAM was significant to their future career. Only a minority reported that CAM was a threat to public health or CAM was in conflict with mainstream biomedicine. The proposal to integrate use of CAM into existing nursing programs received extensive support, with approximately 33.3–93.3% NSs expressing interest and intention to pursue further education and training so that they are equipped to advise patients on the utilization of CAM. Approximately 44.0–98.3% NSs had personal experience with CAM therapy and used it primarily for relaxation and stress relief. Of the wide range of CAM modalities, students had higher self-rated knowledge of massage and herbal medicine. Coincidently, NSs considered these two therapies to be the most effective and the most worthy of recommending to patients.

Twenty-five out of 26 of the studies included were rated to be of moderate to high quality. The aforementioned evidence and results derived from the included studies can therefore be considered credible. All studies used investigator-developed questionnaires/scales to measure NSs' KAP toward CAM, and only a small proportion of these studies reported reliability and validity. Therefore, the development of a standardized and credible questionnaire under current research topic is urgently needed. Overall, the integration of CAM modules into the existing nursing education programs is not only desired and welcomed by a majority of NSs, but also essential for patient safety given nurses are often in the position to advise patients about CAM use.

### Strengths and limitations

To the best of our current knowledge, this is the first systematic review comprehensively investigating the collective evidence regarding the KAP toward CAM among NSs. Among the 26 included studies, the samples originated from 12 countries, covering North America, Europe, Oceania, Africa, and Asia, reflecting the diversity and representativeness of the sample source (Table 1). The quality of this review is further enhanced by the solid academic background of the researchers and multidisciplinary collaboration, with most of our research team members have backgrounds in CAM and/or nursing science, and all members have a dual role of delivering theory at the university and practicing counseling/treatment in the clinical settings.

Yet, despite this review being carried out strictly in accordance with PRISMA guidelines, there are several limitations. First, the review was restricted to articles published in English and Chinese. Given the popularity of CAM modalities in many non-English/non-Chinese speaking countries, for instance Korea, Japan, India, or Iran, it is likely that there are relevant studies published in other languages that may have affected our current findings or led to different conclusions. Actually, during the screening stage, at least eight of the retrieved articles were excluded due to the language limitation (Appendix 4). Second, to reduce heterogeneity across the included studies, we only considered CAM as a broad umbrella term in the literature retrieval, and discarded studies investigating NSs' KAP toward a specific type of CAM, even including many representative CAM modalities with significant national/regional characteristics [e.g., Ayurveda/Unani for Indian NSs, Traditional Chinese Medicine (TCM) for Chinese NSs, osteopathic/chiropractic for Australian/New Zealand/US NSs, or Arabic medicine for Arab states and regions, etc.]. It is possible that those studies would further enrich the results of the current review. We, however, do not think exclusion of those studies would skew the conclusion of this review given we are only interested in the broad concept of CAM internationally, rather than focusing on one specific region. Third, all studies adopted self-administered questionnaire/scale rather than a standardized and uniform instrument, and only 30.8% of these studies tested psychometric properties of the employed instrument. The reliability of the results is thus undermined. Finally, qualitative study (interview) is more appropriate than quantitative study for accessing and interpreting complex attitudes/belief/perceptions. We did not include qualitative studies and as such, it should be acknowledged that our interpretation/understanding of NSs' attitudes toward CAM are somewhat restricted. Further rigorous and well-designed mixed-methods studies (using quantitative questionnaires/scales with good reliability and validity for investigating NSs' KAP and its influencing factors, and using qualitative depth interviews for exploring reasons underlying their attitudes and behaviors) with larger and more diverse samples are required to build stronger and more conclusive evidence.

### A comparison with previous systematic reviews

Two systematic reviews were published in 2017 (2) and 2018 (47), respectively, with the aim of reviewing nurses' KAP toward CAM. The former critically reviewed nurses' attitudes through a meta-synthesis of qualitative research, without covering the knowledge and behavioral dimensions (2). In the latter (47), however, nurse was viewed as a broad concept, including clinical nurses, midwives, NSs and nursing faculty. As a result, there was considerable heterogeneity among the included studies because of the discrepancy in study subjects, which introduces extra variability and makes it difficult to interpret the results. Here, we only targeted NSs to reduce variability and subsequently to better reflect the real KAP level of this population. In the results section, we also summarized the two to three CAM modalities that NSs were most familiar with, had most interests to learn, found most effective, and had most likelihood to recommend to patients. Such information is considered as KAP-related dimensions, which further flesh out our findings. Finally, based on the NSs' general KAP toward CAM reviewed in this study, we also provide a recommendation to adopt teaching strategies regarding introduction/integration of CAM module in the following paragraphs. None of this information was provided in those two previous systematic reviews. It is acknowledged that our major findings are consistent with those of the two reviews. That is, current nurses/future nurses (NSs) have very limited education in CAM and a lack of professional frameworks to assist them in this area.

### Interpretation of findings in NSs' overall KAP levels of CAM

Targeting NSs in the current review is believed to be justified and necessary since the perceptions on CAM amongst the new generation of healthcare workers and professionals are likely to influence the future direction of health care (22), including the development and clinical application of CAM therapies. Moreover, in consideration of the heterogeneity across the studies arising from the research design, particularly the adopted various self-administrated questionnaires/scales in each study, our utilization of a systematic review approach allows for a more comprehensive understanding of the general level of KAP toward CAM amongst NSs.

Nurses are the largest group of healthcare providers across the globe, and the professional recommendation for CAM therapies has been found to be associated with nurses' knowledge background (20). According to the present review, amongst the numerous therapies, massage and herbal medicine are best known to NSs, and also top the list as the two modalities NSs are most likely to be recommend to their future patients. More to the point, the CAM knowledge held by nurses is pivotal in both health promotion and disease prevention (47). In order to manage this role aptly, NSs need to be prepared for their roles from studentship onwards (27, 29). Unfortunately, in light of the evidence collected in this review, it appears that NSs, as future nurses, have limited knowledge of CAM and lack professional frameworks to assist them. NSs' knowledge level of CAM was reported to be higher than that of medical students in two studies (8, 45); and was reported to be not significantly different from that of their counterparts, that is students of other health professions, i.e., medicine, pharmacy, midwifery, health visitor, and chiropractic, etc. in three studies (9, 39, 43). The discrepancy may be attributed to the variations in cultural backgrounds of the respondents and in CAM modalities covered by each different survey. No studies reported that NSs had lower knowledge level of CAM than their counterparts. In line with this, NSs generally had greater likelihood than their counterparts of consulting a CAM practitioner (41), or using/consider using CAM approaches (8, 28, 37). These results suggest that NSs, although with limited knowledge of CAM, are generally comparable or better informed than other health profession students about CAM modalities.

We are not surprised by these findings, as some CAM modalities (e.g., music therapy, simple relaxation therapy, meditation, etc.) in fact are already included in the “Nursing Interventions Classification” developed by the “Iowa Intervention Project” since 1992 and have long been part of nursing practice in some countries (8, 16, 48), as well as being accepted as an independent function used by nurses in patient care (8). Illustratively, the nursing intervention recommended by the North American Nursing Diagnosis Association for diagnosis of “energy field disturbance” includes meditation, acupressure, and simple guided imagery (16, 49). These therapies evidently fall under the umbrella of CAM, whilst they are not labeled as such (16). Some nurses thereby even argue positively that CAM creates an opportunity to reclaim person-centered holistic care and broaden the meaning of contemporary nursing care practice (2). Another possible explanation for NSs show higher CAM knowledge level than medical students is that the latter generally pay less attention than NSs to CAM modalities, as pharmacotherapies occupy the foremost place in the classical understanding of medicine (8). In addition, nurses are more ready to accept patient preferences and autonomy in their own healthcare, which can include CAM therapies that patients report that are effective (2, 10, 50). It is, therefore, clear that the systematic integration of CAM into existing nursing programs is well-grounded in both theory and tradition (16). Furthermore, this kind of pedagogical reform appears to have a positive impact on the knowledge and/or interests of CAM among NSs at any level (43). One strong piece of evidence is that a pedagogical experiment performed in University of Washington has confirmed that following CAM being incorporated into the curricula, 70% of NSs at all levels reported an increase in their CAM knowledge and 50% reported an increase in their level of CAM interests (5). Patients may also benefit from nurses better prepared, as a previous study showed that the greater CAM knowledge NSs possess the more positive they will practice in their clinical area (45).

NSs' attitude toward CAM has been suggested to greatly influence implementation in contemporary nursing practice (46, 51). According to current review, only a low proportion of NSs surveyed viewed CAM as being in conflict with mainstream medicine, or even a threat to public health. Such findings are in line with the conclusion of a prior systematic review focusing on similar theme but allowing nurses as respondents (2). Clearly, regardless of nurses or NSs, their support for CAM is not an attempt to challenge modern medicine but rather an endeavor to further improve the current care quality to their patients *via* the supplement of CAM and supporting patient autonomy and choice (2). Furthermore, despite debate with respect to the attributes of CAM curricula (elective or compulsory) (9, 32, 40), more knowledge of CAM is in general welcomed among NSs, with most of the NSs surveyed reporting an interest in further learning and support for the integration of CAM courses into existing nursing programs (8, 9, 15, 16, 27–33, 35, 37, 38, 41, 44–46). Just as Lindeman declared, nursing education must be broad-based, community-based, keeping a holistic focus, and affording alternatives to Western medicine (52). These findings are encouraging, because research has suggested that NSs' positive attitudes toward CAM can be used to predict their behavior with regard to CAM's use and recommendation to their future patients (13). Conversely, patients' decision concerning the CAM therapies is also likely to be influenced by nurses' attitude toward these therapies (2).

Besides favorable attitudes, the majority of NSs strongly agreed that patients should inform their medical treating team about their use and preference of CAM modalities (13). Such finding also highlights the need for further education on CAM, so that NSs can safely advise their patients both the effectiveness and side-effects of each therapy. Equipped by greater knowledge and more thorough understanding, healthcare practitioners may also discuss with their patients the indications and contraindications of alternate remedy options so that patients can make an informed choice (2). In contrast, lack of qualifications in practicing such CAM modalities, or lack of sufficient knowledge of their effects or even hazards may lead to varied issues for patients (29).

Development and support from faculty is another identified enabler for the integration of CAM into nursing teaching and clinical practice (16). Promisingly, many studies reported that most nursing faculty expressed agreement and desire for more CAM education (15, 16, 37).

Finally, in the current review, knowledge level, previous positive personal experience, and/or personal cultural background (including religious/beliefs) are identified as the potential factors which can influence NSs' attitudes toward CAM. For instance, increased knowledge contributes to the positive attitudes (53). Schramm et al. reported that following participation in a CAM course, nearly all NSs agreed CAM was important and benefits future advanced practice (54). Interestingly, gender appears to be another possible influential factor. Previous studies demonstrated that female students in general had a more positive attitude toward CAM and they were more likely to use CAM in comparison with their counterparts (14, 43). This may enable it easier to deliver CAM education among NSs, since the potential gender lopsided of the nursing profession and the number of female NSs far exceeds that of male NSs.

In current review, the overall rate of personal use of CAM amongst NSs ranged from 44 to 98.3%. The primary purposes for their CAM usage included reduction of stress, relaxation, and health improvement, which are in agreement with the purposes of personally using CAM by nurse reported in a prior systematic review (47). Previous personal experience is one of the most common and critical factors in shaping NSs' attitudes toward CAM (2, 39) and NSs' likelihood of recommending CAM therapies to future customers (55). Sahin et al. (32) also observed a positive correlation between the status of NSs benefitting from CAM and recommending CAM. The same phenomenon has been identified amongst nurse population as well (2). In study of Sohn et al., over 60% nurse acknowledged that they relied on “personal experience” for knowledge about CAM (56). As reported, even amongst those nurses who are previously skeptical about the value of the CAM therapies, they may also attribute the positive experience with their decision to utilize or recommend such therapies to their customers (2). The current review also found that many NSs' supports for CAM therapies were largely based on their previous positive use experience or based on patients' reports. And, both two bases (personal experience and patients' reports) were also judged by NSs as the most two crucial evidence and reference when making clinical decisions regarding CAM, such as using or recommending a specific CAM modality (16, 41).

### Implications for education

Current evidence has critical implications to guide and instruct the CAM courses design, including development of course syllabus, and compilation of teaching materials, for NSs. Several questions associated with curriculum such as “What to teach,” “Who can teach,” and “How to teach” may also be informed by the results we summarized in this review.

#### Answers to “what to teach”

##### Using evidence as a core to select what to deliver and teach

Curricula content about CAM is recommended to be divided into two sections, namely broad foundational skills and specific content areas (57). Introductory foundational principles should be delivered first, aiming to provide NSs a framework for future learning (57). Such principles are advised to cover at least the CAM professional terms, frequent reasons for CAM use, the basic tenets of CAM healing systems, and skills for initiating dialogue (including enquiry and discussion) of CAM with patients in a non-judgmental manner (57). There is a wide variety of CAM modalities available, and which of these should be prioritized for inclusion in syllabuses and textbooks for specific content section requires discretion. Scientific evidence is central to both healthcare practice and medical education, and CAM must be held to similar standards (57, 58). Hence, the scientific evidence for or against the effectiveness and safety of each CAM modality must be at the center of selecting what to deliver and teach (57). To ensure the updating of knowledge and the sustainability of the curricula, curricula developers should also consider the need for constant review and critical evaluation as curricula-related new CAM evidence emerges (59).

NSs' interest, attitude and level of knowledge can be viewed as other valuable basis and reference. In accordance with current evidence, we recommend that massage, nutritional/diet-based therapy (including vitamins, minerals, and other supplements), music therapy, meditation, aromatherapy, phytotherapy/herbal medicine, and therapeutic/healing touch be programed into the CAM curricula, considering these modalities have been deemed by NSs as the ones they have most interests in. In fact, these modalities cover almost all of the top three modalities that NSs know best, find most effective, and personally use most frequently. Educators are also advised to give consideration to numerous variables regarding use of each CAM modality, including regional beliefs, cultural preferences, traditional practices, economic and income status, and clinical applicability/usefulness when designing course (32). Moreover, these variables should first be investigated and examined in the country and region (32) where the course is delivered/taught, so as to determine if a particular modality is eligible to be compiled into the teaching materials. To exemplify, for Asian countries such as India, Korea, Japan, and China, priority may be given to acupuncture; for Western countries, priority could be given to nutrition and CAM manual therapies such as massage, chiropractic and osteopathy; for religious state, priority may be given to prayer, spirituality, or spiritual healing.

##### Enhancing knowledge and skill training of evidence-based practice in CAM curricula

Generally in keeping with findings from nurses (47), insufficiency of scientific evidence was also perceived by NSs as one of the biggest barriers to the usage and integration of CAM. These findings are reassuring in that they reflect the great importance NSs attach to evidence-based practice (EBP) concepts and principles. This is extremely valuable because some CAM therapies such as TCM (60, 61) or Ayurveda (62), despite their popularity and prosperity in China and India, respectively, rely heavily on the practical wisdom inherited from ancient practitioner, rather than high-quality scientific research evidence. In fact, the national nursing education policy recently issued in China has made it explicit that EBP should be one of the basic capabilities for nursing undergraduates (including TCM nursing majors), and that TCM nursing discipline can be advanced through the implementation of EBP (60). Takata et al. also suggest that nurses who have been educated about the scientific evidence for CAM may play critical roles in advising on CAM, including its benefits and risks, as well as in supporting the self-care in response to patients' clinical requirements (63). Meanwhile, these roles would also strengthen the relationship between nurses and their patients, which, in turn, enhance the professional motivation of nurses (63). Therefore, we also encourage enhanced training on EBP knowledge and skills in CAM curricula so that NSs are likely to transform CAM nursing care from praxis-based to evidence-based, and consequently, implementing and optimizing evidence-based CAM care in their future professional career (60). Correspondingly, the methodology and the tools used in the evaluating quality of evidence are ought to be introduced or strengthened. Such a course design should make sense. After all, the study by André et al. has confirmed that embedding evidence implementation into programs did improve NSs' EBP capacity (64). Zhou et al. even provided nursing educators with more specific and detailed advice, that is, course syllabuses could be designed on the basis of comprehensive EBP models with five steps, such as the JBI Model of Evidence-Based Healthcare, and the ACE-star model (60).

##### Incorporating literature/information retrieval skill training into CAM curricula

To avoid harmful practices and reduce associated risks, it is essential that healthcare workers have reliable and accessible information and/or evidence (31). Specifically, healthcare professionals including nurses should be capable of delivering research-based CAM information to their patients (17, 65), and/or guiding their patients to identify the reliable and professional CAM information from the clutter (10). Currently, NSs generally lack adequate information about CAM (31). Also, high-quality sources of information, that is, evidence-based textbooks, clinical guidelines, or expert's consensus, etc. were not the primary source for NSs collecting CAM information. Our review revealed that the most common sources that NSs obtained CAM information were newspaper/magazines/TV and internet, and lower proportion had sourced CAM information from professional healthcare providers or scholastic education. This contrasts to a prior systematic review, in which professional colleagues and journals were the main sources of CAM information for practicing nurses (47). In some regions, apparently, NSs may have no access to credible sources and may have to rely on media and internet to gain relevant information. Yet, the accuracy and reliability of such informal information can be a concern and should be questioned (17, 29). Social media may even contain misleading anecdotal information that promotes unscientific therapies (36, 66). Such scenario also hints at the serious neglect of teaching NSs how to access and screen reliable CAM information in existing nursing education.

These findings lead to the strong recommendation of incorporating literature/information retrieval content of CAM therapies into nursing curricula, in order to help NSs learn to access scientific, accurate, and reliable CAM information from specialist medical databases. We are confident in the feasibility of this recommendation because a prior study has confirmed that nurses who had previously received CAM-related education were three times more likely to seek CAM information from a medical database than those without (20). Another reason that explains our emphasis on CAM-related retrieval skill is that it can be perceived as a life-long learning strategy or “long-lasting tools” to help NSs better cope with the current and emerging CAM-related therapies that they may encounter in their future clinical work (57). After all, regardless of how much content is delivered in a curriculum, science keeps evolving and questions keep changing. With capability to easily source evidence-based materials, NSs will know where to find reliable and up-to-date evidence/information concerning CAM, and know how to better objectively discriminate, judge, assess, and interpret those retrieved results (57). Accordingly, some practical databases that facilitate students' search for high-quality literature and books related to CAM therapies should be introduced to support the curricula.

#### Answers to “who can teach”

The lack of teaching staff and their professional training is considered to be one of the most significant barriers to the use of CAM in clinical settings (15). Due to the integrated nature of the CAM curricula, staff usually find it difficult to teach individually and to estimate the amount of time devoted to the teaching work (67). So, who are the most suitably qualified staff? It is suggested that the teaching staff can consist of two types of professionals: First, industry practitioners/community-based practitioners with a full educational background (from undergraduate to PhD) in CAM. Second, registered nurses with enough experience in education/training and clinical practice in CAM. Furthermore, the teaching tasks of these two types of professionals are suggested to be focused. Industry practitioners can lead the delivery of theoretical lectures. And, different CAM therapies should be taught by the corresponding practitioners to ensure the professionalism and teaching quality (e.g., chiropractic/osteopathy is best to be taught by chiropractor/osteopath, TCM/acupuncture is best to be taught by TCM practitioner/acupuncturist, etc.). In contrast, the clinical placements should be led by the registered nurses with extensive CAM experience. Having a nurse as a “peer predecessor” to lead the placement might help NSs quickly adapt to clinical work and better translate theory into practice, so that they can be truly equipped to provide reliable CAM-related advice, treatment or referral services to patients in need. Although both industry practitioners and registered nurses might introduce an element of bias, this could be overcome with an evidence-based teaching method and clear learning outcomes (59). Notably, in view of sustainability, the qualifications of these staff for such curricula must be consistent and their repeated involvement sustainable (59).

#### Answers to “how to teach”

##### Specialization determines the nature (compulsory/elective) of the curricula

There is still debate as to whether the CAM curricula should be delivered as an elective or a compulsory module (9, 32, 40). None of the included studies explored this issue. Whereas, inferences made by some authors in the discussion section may provide a few potential clues. For instance, proponents for “compulsory module” might reckon that being taught in a compulsory rather than an elective format would help NSs to overcome the CAM knowledge gap more comprehensively and effectively (40). James et al. suggest one possible reason for those who prefer CAM as an elective to a compulsory module is that the NSs interviewed are already overstretched with their major courses and may view a compulsory CAM module as an added burden (9). It should be stated that the respondents in James' survey were all final year undergraduate students (9). Thus, it is understandable that the extra burden of a compulsory CAM course in last year is unwanted by students nearing graduation.

A compulsory course is a curriculum within the program that must be completed in order to fulfill the requirements to be eligible to graduate; elective course is one which the students can opt into if they so desire and thus are usually more interested and motivated in learning and enriching their professional portfolio (68). It is limiting to discuss the nature/format of a curriculum outside of the disciplinary context, and it may be feasible to defuse this dispute through developing various specializations. In Hong Kong Metropolitan University, Bachelor of Nursing includes three specializations (Bachelor of Nursing, Bachelor of Nursing in General Health Care, and Bachelor of Nursing in Mental Health Care) for potential students to choose^1^. Similarly, in University of South Australia, students who are seeking study in Graduate Certificate in Nursing can choose one specialism from several specializations (e.g., Cardiovascular Nursing, Critical Care Nursing, Health and Aging, Nurse Education, etc.)^2^. With reference to the mode of these majors, we suggest that CAM curricula should be a compulsory module for Bachelor/Diploma/Certificate/Master of Nursing (in CAM specialization), while be an elective module for other Nursing degrees.

##### Clinical placements and experiential learning mode are strongly encouraged

Given NSs/nurses are used to judging or recommending CAM modalities to patients based on their previous personal experience (which was detailed in the previous paragraph “section Interpretation of Findings in NSs' Overall KAP Levels of CAM”), incorporating clinical placements to match theoretical knowledge (formal lectures or tutorials) is strongly encouraged in CAM curricula (33). Clinical placements provide NSs with learning opportunities that may affect their opinions on CAM practices for patients (33). Experience derived from clinical placements may benefit NSs to better perform their patient assessment and patient engagement capabilities which would include identification of the patients' utilization of CAM therapies, the effects thereof, and keeping the patients and the families informed of these practices (33). Hence, practicing communication and critical thinking skills about CAM therapies and the prevalence of their use should be highlighted in the clinical placements teaching (21).

Additionally, we also strongly recommend the inclusion of experiential learning mode (15, 16), involving activities that include personal participation in self-care activities (15). It is clear that experiential learning will be favored compared to a solely didactic approach, as such immersive approach can significantly enhance student engagement (59). Many mind-body techniques are well-suited and encouraged to being taught in the form of experiential learning mode (57). As an illustration, mindfulness meditation impacts positively on NSs' anxiety, depression, stress, burnout, and sense of wellbeing (69). The same goes for the benefits of yoga (70). Delivering this species of therapeutic remedy *via* experiential learning mode thereby can serve the dual purposes of benefiting the NSs at present and, possibly, their patients in the near future (57). All such designs and ideas mentioned above are not only expected to facilitate the clinical translation and application of NSs' theoretical knowledge of CAM, but are also expected to facilitate them to be more confident in providing unbiased advice to patients in their future career.

### Implications for research

#### Development and test of the professional instrumentation

A crucial research gap of concern is a lack of established instrumentation that can be used to evaluate NSs' KAP toward CAM. Two included studies (9, 39) employed CHBQ, which is a questionnaire for measuring medical students' attitudes/beliefs toward CAM (71). Despite the potential adaptability of CHBQ to other health professions education settings (71), those aforementioned two studies did not re measure its psychometrics (reliability and validity) when adapting the CHBQ (9, 39). Two studies (27, 29) used HCAMQ, which includes 11 questions [six questions are relevant to beliefs about the scientific validity of CAM (CAM subscale), and the remaining five are relevant to beliefs about holistic health (holistic health subscale)] (72). Re-test reliability of the total HCAMQ and CAM subscale was 0.86 and 0.82, respectively (72). Both aforementioned studies adopted total HCAMQ rather than CAM subscale, and calculated the internal consistency and homogeneity between the items, with Cronbach's coefficient alpha were 0.72 (27) and 0.74 (29), respectively. Another study (36) employed ACTS to determine healthy students' attitude toward using CAM therapies (Cronbach's alpha coefficient 0.79). In that study, the Cronbach's alpha coefficient was 0.84 (36). All these three instrumentations are intended to examine attitudes/belief toward CAM only, and do not cover dimensions link to knowledge and behavior/practice nor are they specific to NSs as respondents. Hence, the urgent need remains to carefully develop a practical, valid and reliable instrumentation for assessing NSs' KAP toward CAM.

To better promote and deliver CAM education amongst NSs, evaluation related to effectiveness of this knowledge applied to clinical practice is also necessary. To match this, it is also pivotal to develop and validate methods for measuring other instructional outcomes such as CAM knowledge and skill acquisition amongst NSs at the end of a CAM specific course if that is the chosen model used to embed in the curricula (71).

#### A need in investigating Chinese NSs' KAP toward CAM

A previous review indicated that nurses from China and US had a more positive attitude toward CAM use than did nurses from other countries (47). We however found no surveys available in the databases that investigated Chinese NSs' KAP toward CAM, in spite of a comprehensive search. Due to the popularity of TCM in China and the labor demand of massive TCM hospitals, many universities and colleges in China (particularly TCM universities) introduce TCM nursing curricula into nursing programs at all levels, covering diploma, undergraduate, and/or postgraduate (73). Whilst many Chinese use other CAM modalities such as yoga, aromatherapy, or massage [non-TCM massage (Tuina)], knowledge of these modalities is hardly taught at any level of nursing education, to our best knowledge. In consequence, clinical practitioners may find it difficult when patients reveal information about preferences and previous utilization of CAM therapies other than TCM (74). These findings imply that there is an urgent need to investigate Chinese NSs' KAP toward different CAM modalities and introduce CAM modules other than TCM into existing nursing education accordingly.

## Conclusions

The current review identifies a need to enhance the skills to discriminate evidence-based CAM therapies by NSs through integrating CAM modules into existing nursing programs, in order to prepare future nurses to competently and safely advise patients use and to enhance safe and patient-centered care. In curricula design, the scientific evidence for or against the efficacy and safety of each CAM modality should be considered. Experiential learning is a highly recommended educational mode in the curricula for delivering specific CAM modalities. In addition to theoretical knowledge and matched clinical placement, skills training on CAM-related literature search and EBP are also advised to be incorporated into the curricula. From a clinical perspective, such a teaching model that well bridge theory to clinical practice, will allow for evidence-based practice. To help reduce costs and increase the quality of research associated with NSs' KAP toward CAM, a standard, comprehensive, and applicable instrumentation should be designed and examined for utilization in different cultures.

## Data availability statement

The original contributions presented in the study are included in the article/Supplementary materials, further inquiries can be directed to the corresponding author/s.

## Author contributions

ZZ, GK, and Q-QF conceptualized and designed this review. W-JZ, Q-QF, and F-YZ performed database search, as well as data extraction, analysis, and interpretation and were also involved in the quality assessment. F-YZ drafted the research protocol and prepared the manuscript. RC, SC, GK, and ZZ provided critical comments for revising the manuscript. All authors have read and approved the final version.

## Funding

This work was sponsored by RMIT Research Stipend Scholarship from RMIT University Australia, University's Scientific Research Project at Shanghai Sanda University [2021zz02-yj] Shanghai, China to F-YZ, and Foreign Graduate Student Education Research Project at Shanghai University of Traditional Chinese Medicine Shanghai, China (JX211072N and IEC2021YJSDS11) to W-JZ.

## Conflict of interest

The authors declare that the research was conducted in the absence of any commercial or financial relationships that could be construed as a potential conflict of interest.

## Publisher's note

All claims expressed in this article are solely those of the authors and do not necessarily represent those of their affiliated organizations, or those of the publisher, the editors and the reviewers. Any product that may be evaluated in this article, or claim that may be made by its manufacturer, is not guaranteed or endorsed by the publisher.

## Footnotes

^1^ https://www.hkmu.edu.hk/nhs/programmes-courses/nursing-and-health-studies/undergraduate-programmes/

^2^ https://study.unisa.edu.au/nursing-midwifery/
