# Supplementary material for: Knowledge about, attitude toward, and practice of complementary and alternative medicine among nursing students: A systematic review of cross-sectional studies
Source: Front Public Health. 2022 Aug 4;10:946874. doi: 10.3389/fpubh.2022.946874 (PMC9386551; doi:10.3389/fpubh.2022.946874)
Supplement: Supplementary file 1 [file Data_Sheet_1.pdf]

Appendices

Appendix 1 Categories of CAM modalities

**Nutritional therapies**	**Psychological therapies**	**Physical therapies**	**Combinations such as psychological and physical or psychological and nutritional therapies**	**Other Complementary Health Approaches**
● Special diets<br>● Dietary supplement<br>● Vitamins and minerals<br>● Herbs (Botanicals)<br>● Probiotics<br>● Microbial-based therapies	● Meditation<br>● Hypnosis<br>● Music therapies<br>● Relaxation therapies (e.g., breathing exercises, guided imagery)<br>● spiritual practice<br>● Mindfulness-based stress reduction<br>● Trager psychophysical integration	● Acupuncture<br>● Massage<br>● Spinal manipulation<br>● Chiropractic manipulation<br>● Osteopathic manipulation	● Yoga<br>● Tai-chi<br>● Dance therapies<br>● Some forms of art therapy<br>● Mindful eating<br>● Qi-gong<br>● Pilates	● Traditional healers<br>● Ayurvedic medicine<br>● Traditional Chinese medicine<br>● Homeopathy<br>● Naturopathy<br>● Functional medicine

Abbreviations: CAM, complementary and alternative medicine

Appendix 2 List of search terms

Population	Context	Outcomes
“nursing student”	“complementary and alternative medicine”	“knowledge”
“student nurse”	“complementary medicine”	“perception”
	“complementary therapy”	“attitude”
	“alternative medicine”	“belief”
	“alternative therapy”	“use”
	“natural medicine”	“practice”
	“natural therapy”	“experiences”
	“naturopathy”	“KAP”
	“integrative medicine”	
	“integrative therapy”	

Appendix 3 Search strategy for each database

Databases	Search strategy
MEDLINE (via PubMed)	#1 nursing student[MeSH Terms]<br>#2 nursing student[Title/Abstract]<br>#3 student nurse[Title/Abstract]<br>#4 #1 or #2 or #3<br>#5 complementary medicine[MeSH Terms]<br>#6 complementary therapy[MeSH Terms]<br>#7 complementary therapy[Title/Abstract]<br>#8 complementary medicine[Title/Abstract]<br>#9 natural therapy[Title/Abstract]<br>#10 natural medicine[Title/Abstract]<br>#11 integrative medicine[Title/Abstract]<br>#12 integrative therapy[Title/Abstract]<br>#13 #5 or #6 or #7 or #8 or #9 or #10 or #11 or #12<br>#14 knowledge[MeSH Terms]<br>#15 knowledge[Title/Abstract]<br>#16 perception[MeSH Terms]<br>#17 perception[Title/Abstract]<br>#18 attitude[MeSH Terms]<br>#19 attitude[Title/Abstract]<br>#20 belief[Title/Abstract]<br>#21 practice[Title/Abstract]<br>#22 experiences[Title/Abstract]<br>#23 use[Title/Abstract]<br>#24 KAP[Title/Abstract]<br>#25 #14 or #15 or #16 or #17 or #18 or #19 or #20 or #21 or #22 or #23 or #24<br>#26 #4 and #13 and #25
EMBASE (via Ovid)	#1 exp nursing student/<br>#2 nursing student.ab,ti.<br>#3 student nurse.ab,ti.<br>#4 #1 or #2 or #3<br>#5 exp alternative medicine/<br>#6 complementary medicine.ab,ti.<br>#7 complementary therapy.ab,ti.<br>#8 natural medicine.ab,ti.<br>#9 natural therapy.ab,ti.<br>#10 exp integrative medicine/<br>#11 integrative medicine.ab,ti.<br>#12 integrative therapy.ab,ti.<br>#13 #5 or# 6 or #7 or #8 or #9 or #10 or #11 or #12<br>#14 exp knowledge/

	#15	knowledge.ab,ti.
	#16	exp perception/
	#17	perception.ab,ti.
	#18	attitude.ab,ti.
	#19	exp attitude/
	#20	belief.ab,ti.
	#21	exp practice act/
	#22	practice.ab,ti.
	#23	experiences.ab,ti.
	#24	exp experience/
	#25	KAP.ab,ti.
	#26	#14 or #15 or #16 or #17 or #18 or #19 or# 20 or# 21 or #22 or# 23 or #24 or #25
	#27	#4 and #13 and #26
Nursing & Allied Health Database (ProQuest)	#1	AB,TI(nursing student)
	#2	mesh(students, nursing)
	#3	AB,TI(student nurse)
	#4	#1 or #2 or #3
	#5	mesh(complementary therapies)
	#6	mesh(integrative medicine)
	#7	AB,TI(complementary medicine)
	#8	AB,TI(complementary therapy)
	#9	AB,TI(natural therapy)
	#10	AB,TI(natural medicine)
	#11	AB,TI(integrative medicine)
	#12	AB,TI(integrative therapy)
	#13	#5 or #6 or #7 or #8 or #9 or #10 or #11 or #12
	#14	mesh(knowledge)
	#15	mesh(perception)
	#16	mesh(attitude)
	#17	AB,TI(knowledge)
	#18	AB,TI(perception)
	#19	AB,TI(attitude)
	#20	AB,TI(belief)
	#21	AB,TI(practice)
	#22	AB,TI(experiences)
	#23	AB,TI(use)
	#24	AB,TI(KAP)
	#25	#14 or #15 or #16 or #17 or #18 or #19 or #20 or #21 or #22 or #23 or #24
	#26	#4 AND #13 AND #25
AMED: Allied and Complementary Medicine Database	#1	student nurses or nursing students or student nurse or nursing student or undergraduate nurse
	#2	complementary therapies or alternative therapies or nonpharmacological therapy
	#3	nature therapy or ecotherapy or forest therapy or nature based therapy
	#4	integrative medicine or complementary medicine or alternative medicine
	#5	#2 or #3 or #4

	#6 knowledge<br>#7 perceptions or attitudes or opinion or experience or view or reflection or beliefs<br>#8 practice<br>#9 experiences<br>#10 use<br>#11 KAP<br>#12 #6 or #7 or #8 or #9 or #10 or #11<br>#13 #1 and #5 and #12
China National Knowledge Infrastructure (CNKI)	(篇关摘=护生) OR (篇关摘=护理学生) AND (篇关摘=补充医学) OR (篇关摘=补充疗法) OR (篇关摘=替代医学) OR (篇关摘=替代疗法) AND (篇关摘=知信行) OR (篇关摘=知识) OR (篇关摘=态度) OR (篇关摘=行为)
Wanfang database	题名或关键词:(护生) and 题名或关键词:(补充医学) and 题名或关键词:(替代医学) or 题名或关键词:(知识) or 题名或关键词:(态度) or 题名或关键词:(行为)
Chongqing VIP database (CQVIP)	题名或关键词=护生 AND 题名或关键词=补充替代医学+补充与替代医学+补充和替代医学+补充替代疗法 AND 题名或关键词=知识 OR 题名或关键词=态度+信念 OR 题名或关键词=行为+实践
China biomedical literature service system (SinoMed)	"护生"[标题:智能] AND "补充医学"[标题:智能] OR "替代医学"[标题:智能] OR "补充疗法"[标题:智能] OR "替代疗法"[标题:智能] AND "知信行"[标题:智能] OR "知识"[标题:智能] OR "态度"[标题:智能] OR "行为"[标题:智能]

Appendix 4 Excluded studies with detailed reasons of irrelevance

References	Reasons for exclusion
Aktas B. Attitudes of nursing students toward holistic complementary and alternative medicine. *JAREN*, 2017; 3(2):55-59.	non-English/non-Chinese literature
Altinbas Y, Ister ED. Opinions, information and applications about complementary and alternative therapies of health school students. *Sak Univ J Holist Health*, 2019; 2(1):47-60.	non-English/non-Chinese literature
Araz Col N, Tasdemir HS, Kilic Parlar S. Evaluation of opinions of the faculty of health sciences students about non medical alternative and traditional therapies. *Gumushane University J Health Sci*, 2012; 1:239-251.	non-English/non-Chinese literature
Aslan H, Unsal A. nursing students' perception levels of spirituality and spiritual care in Turkey. *J Relig Health*, 2021; 60(6):4316-4330.	focus on specific CAM modality/modalities
BAĞCıH, YüCEL ŞC, BAŞAK ME. Examination of the level of knowledge nursıng students regardıng therapeutıc touch. *Progress in Health Sciences*, 2021; 11(2): 1-9.	focus on specific CAM modality/modalities
Baltaci N, Koc E. Knowledge, use and attitude of intern nursing and midwifery students with regard to complementary and alternative medicine. *Samsun Sağ Bil Der*, 2018; 3(1):10-16.	mixed results for nursing students with other profession (midwifery); the lack of separate data for nursing students
Booth-Laforce C, Scott CS, Heitkemper MM, Cornman BJ, Lan MC, Bond EF, Swanson KM. Complementary and Alternative Medicine (CAM) attitudes and competencies of nursing students and faculty: results of integrating CAM into the nursing curricula. *J Prof Nurs*, 2010; 26(5):293-300.	incompatible with the purpose of current review
Cai Y, Boyd DL. Effect of a traditional Chinese medicine course for undergraduate nursing students: A pre-/post-test study. *Nurse Educ Today*, 2018; 70:87-93.	incompatible with the purpose of current review
Camurdan C, Gul A. Complementary and alternative medicine use among undergraduate nursing & midwifery students in Turkey. *Nurse Educ Pract*, 2013; 13(5):350-354.	mixed results for nursing students with other profession (midwifery); the lack of separate data for nursing students
Chung MS. Nursing students' attitude towards complementary and alternative therapies and their curricula expectations regarding it. *J Korean Acad Soc Nurs Edu*, 2012; 18(2): 188-196.	non-English/non-Chinese literature
Doğanay S. Complementary and alternative medicine: understanding, attitude and usage among Turkish health sciences and medical students. *J Contemp Med*, 2018; 8(1):00-00.	without target research subjects
Fenton MV, Morris DL. The integration of holistic nursing practices and complementary and alternative modalities into curricula of schools of nursing. *Altern Ther Health Med*, 2003; 9(4):62-67.	without target research subjects

Ferreiro CB, Matikainen D, Põldmaa K. Complementary and Alternative medicine from the nursing student's perspective. *Bachelor thesis*, 2019; Arcada University of Applied Sciences.	review; non-cross-sectional quantitative study
Garrett-Wright D, Lartey G, Sturgeon LP. Kentucky school nurses' knowledge and perceptions of complementary, alternative and integrative therapies. *Kentucky Nurse*; 67(1): 12-13.	without target research subjects
Groft JN, Kalischuk RG. Nursing students learn about complementary and alternative health care practices. *Complement Health Pract Rev*, 2005; 10(2): 133-146.	incompatible with the purpose of current review
Guzel D, Doganay S, Öztürk D, Tanyeli A. The effects of complementary and alternative medicine on physiological systems: perceptions of health sciences and medical students. *Psychology Research*, 2017; 7(6): 305-317.	without target research subjects
Hon KL, Twinn SF, Leung TF, Thompson DR, Wong Y, Fok TF. Chinese nursing students' attitudes toward traditional Chinese medicine. *J Nurs Educ*, 2006; 45(5):182-185.	focus on specific CAM modality/modalities
Hooshangi M, Mohammadi S, Alizadeh J, Mohammadi M, Bolghanabadi A, Rahmani M, Mansouri S, Mohammadzadeh F. Knowledge, attitude and practice of students of Gonabad university of medical sciences toward famous methods of complementary and alternative medicine. *Trad Integr Med*, 2017; 2(2): 67-73.	mixed results for nursing students with other profession; the lack of separate data for nursing students
Huang RP, Su ZY. The knowledge, attitudes, and behaviors related to gua-sha therapy among college-level nursing students. *Journal of Nursing and Healthcare Research*, 2015; 11(4): 287-297.	focus on specific CAM modality/modalities
Jakovljevic MB, Djordjevic V, Markovic V, Milovanovic O, Rancic NK, Cupara SM. Cross-sectional survey on complementary and alternative medicine awareness among health care professionals and students using CHBQ questionnaire in a Balkan country. *Chin J Integr Med*, 2013; 19(9):650-655.	without target research subjects
Joudrey R, Gough J. Student nurses' use and perceptions of alternative medicine: an exploratory study. *Can J Nurs Res*, 2003; 35(3):80-93.	qualitative research; non-cross-sectional quantitative study
Joudrey R, McKay S, Gough J. Student nurses' perceptions of alternative and allopathic medicine. *West J Nurs Res*, 2004; 26(3):356-366.	qualitative research; non-cross-sectional quantitative study
Keimig T, Braun C. Student nurses' knowledge and perceptions of alternative and complementary therapies. *Journal of Undergraduate Nursing Scholarship*, 2004; 6(1): 7.	no access to full text
Kessack M. Teaching complementary and alternative therapies to prelicensure nursing students: A faculty assessment using a basic qualitative research approach. *Doctorate thesis*, 2015; Capella University.	incompatible with the purpose of current review
Kim do Y, Park WB, Kang HC, Kim MJ, Park KH, Min BI, Suh DJ, Lee HW, Jung SP, Chun M, Lee SN. Complementary and alternative medicine in the undergraduate medical curricula: a survey of Korean medical schools. *J Altern Complement Med*, 2012; 18:870-874.	without target research subjects

Kim YH. A study on nursing students' attitudes toward complementary and alternative medicine (CAM). *J Korea Community Health Nursing Academic Society*, 2004; 18(2): 176-285.	non-English/non-Chinese literature
Kinchen EV, Loerzel V. Nursing students' attitudes and use of holistic therapies for stress relief. *J Holist Nurs*, 2019; 37(1):6-17.	qualitative research; non-cross-sectional quantitative study
Khorasgani SR, Moghtadaie L. Investigating knowledge and attitude of nursing students towards Iranian traditional medicine-case study: universities of Tehran in 2012-2013. *Glob J Health Sci*, 2014; 6(6):168-177.	focus on specific CAM modality/modalities
Lartey G, Sturgeon LP, Garrett-Wright D, Kabir UY, Eagle S. A survey of school nurses' perceptions of complementary, alternative, and integrative therapies. *J Sch Nurs*, 2019; 35(4):256-261.	without target research subjects
Lewith GT, Owen D. Complementary medicine: the Southampton undergraduate experience. *Complement Ther Med*, 2000; 8(3):202-206.	incompatible with the purpose of current review
Lim SH, Lee JY. Nursing students' perception, experience and attitude on complementary and alternative therapies. *Journal of East-West Nursing Research*, 2015; 21(2): 110-118.	non-English/non-Chinese literature
Liu MA, Huynh NT, Broukhim M, Cheung DH, Schuster TL, Najm W. Determining the attitudes and use of complementary, alternative, and integrative medicine among undergraduates. *J Altern Complement Med*, 2014; 20(9):718-726.	incompatible with the purpose of current review
Mei-Ying C, Huey-Shyan L, Chin-Fan T. Student nurses' knowledge, attitude, and behavior toward Chinese medicine and related factors. *J Nurs Res*, 2004; 12(2):103-118.	focus on specific CAM modality/modalities
Melland HI, Clayburgh TL. Complementary therapies: introduction into a nursing curricula. *Nurse Educ*, 2000; 25(5):247-250.	incompatible with the purpose of current review
Nguyen J, Liu MA, Patel RJ, Tahara K, Nguyen AL. Use and interest in complementary and alternative medicine among college students seeking healthcare at a university campus student health center. *Complement Ther Clin Pract*, 2016; 24:103-108.	without target research subjects
North S, Beck B, Liveris M, Vega A, Boyington N, Stockwell L, St George TE, Hopp J. Students' knowledge and self-perceptions regarding integrative medicine and health following training in first-year graduate PA, PT, and OT programs. *J Allied Health*, 2018; 47(3):e91-e95.	without target research subjects
Reed FC, Pettigrew AC, King MO. Alternative and complementary therapies in nursing curricula. *J Nurs Educ*, 2000; 39(3):133-139.	incompatible with the purpose of current review
Richardson SF. Complementary health and healing in nursing education. *Journal of Holistic Nursing*, 2003; 21(1): 20-35.	incompatible with the purpose of current review
Şahin N, Aydın D, Akay B. The attitudes of nursing students towards holistic complementary and alternative medicine. *Balikesir Health Sci J*, 2019; 8(1):21-26.	non-English/non-Chinese literature
Schramm J, Uranga T, Birkle A, Thorp R, Taylor L. Improving holistic care skills for advanced practice registered nurses: Integration of a complementary and alternative medicine course into a doctor of nursing practice curricula. *J Dr Nurs Pract*, 2021: JDNP-D-20-00077.	intervention research; non-cross-sectional quantitative study
Stephenson NL, Brown ST, Handron D, Faser K. Offering an online course: complementary and alternative therapies in nursing practice. *Holist Nurs Pract*, 2007;	incompatible with the purpose of current review

21(6):299-302.	
Subramanian K, Midha I. Prevalence and perspectives of complementary and alternative medicine among university students in Atlanta, Newcastle upon Tyne, and New Delhi. *Int Sch Res Notices*, 2016; 2016:9309534.	incompatible with the purpose of current review
Swanson B, Zeller JM, Keithley JK, Fung SC, Johnson A, Suhayda R, Phillips M, Downie P. Case-based online modules to teach graduate-level nursing students about complementary and alternative medical therapies. *J Prof Nurs*, 2012; 28(2):125-129.	intervention research; non-cross-sectional quantitative study
Takata T, Kuramoto M, Imamura M, Kishida S, Yasui T. Differences in knowledge of and attitudes regarding complementary and alternative medicine among health care profession students in Japan. *JJCAM*, 2013; 10(2): 87-97.	mixed results for nursing students with other profession (midwifery); the lack of separate data for nursing students
Toygar I, Hancerlıoglu S, Gul I, Yondem S, Yilmaz I. Effect of educational intervention on nursing students' attitudes toward complementary and alternative therapies. *International Journal of Caring Sciences*, 2020; 13(2): 1305-1312.	intervention research; non-cross-sectional quantitative study
Trovo MM, da Silva MJ, Leão ER. Alternative/complementary therapies in public and private education: analysis of knowledge among nursing students. *Rev Lat Am Enfermagem*, 2003; 11(4):483-489.	non-English/non-Chinese literature
Weber JP. Nursing students' opinions about acupuncture and Chinese medicine. *Nurs Res*, 1975; 24(3):205-206.	Focus on specific CAM modality/modalities
Wu LF, Liao YC, Yeh DC. Nursing student perceptions of spirituality and spiritual care. J Nurs Res, 2012; 20(3):219-227.	Focus on specific CAM modality/modalities
